# 基于"分子分期"的局部晚期非小细胞肺癌"个体化外科治疗"的长期生存结果

**DOI:** 10.3779/j.issn.1009-3419.2011.02.15

**Published:** 2011-02-20

**Authors:** 清华 周, 应康 石, 军 陈, 斌 刘, 允 王, 大兴 朱, 洪涛 张, 鹏 徐, 友陵 宫, 钢 陈, 森 韦, 小明 邱, 中喜 牛, 晓峰 陈, 哲 雷, 亮 段, 伫 伍

**Affiliations:** 1 300052 天津，天津医科大学总医院肺癌研究所，天津市肺癌转移与肿瘤微环境重点实验室 Tian Key Laboratory of Lung Cancer Metastasis and Tumor Microenvironment, Tianjin Lung Cancer Institute, Tianjin Medical University General Hospital, Tianjin 300052, China; 2 610041 成都，四川大学华西医院胸外科 Department of Cardiothoracic Surgery, West China Hospital, Sichuan University, Chengdu 610041, China; 3 215123 苏州，苏州大学癌 症分子遗传学实验室 Suzhou University Laboratory of Cancer Molecular Genetics, School of Basic Medicine and Biological Sciences, Medical College of Soochow University, Suzhou University, Suzhou 215123, China; 4 200433 上海，上海同济大学附属上海市肺科医院胸外科 Department of Toracic Surgery, Shanghai Pulmonary Hospital, Shanghai Tongji University, Shanghai 200433, China

**Keywords:** 局部晚期非小细胞肺癌, 微转移分子诊断, 分子分期, 个体化外科治疗, Locally advanced non-small cell lung cancer, Micrometastasis molecular diagnosis, Molecular staging, Personalized surgical treatment

## Abstract

**背景与目的:**

局部晚期肺癌约占肺癌的35%-40%，其内科治疗平均生存6-8个月。本研究的目的是探讨和总结检测肺癌病人外周血"微转移"，对局部晚期肺癌病人进行"分子分期"，指导选择局部晚期肺癌手术适应症、术前新辅助化疗和术后辅助治疗的受益者，以及对局部晚期肺癌进行"个体化外科治疗"的可行性及其对长期生存的影响。

**方法:**

应用RT-PCR检测516例局部晚期肺癌病人手术前外周血CK19 mRNA表达（其中115例行术前新辅助化疗者在新辅助化疗前后分别检测病人外周血"微转移"），对肺癌"微转移"进行"分子诊断"，对肺癌病人进行"个体化分子分期"，指导临床选择外科手术适应症、术前新辅助化疗和术后辅助治疗的获益者。回顾分析516例局部晚期肺癌基于"分子分期"的"个体化外科治疗"的长期生存结果。

**结果:**

516例病人中鳞癌322例，非鳞癌194例；P-TNM分期：ⅢA期112例，ⅢB期404例；"个体化分子P-TNM分期"：M-ⅢA期：97例，M-ⅢB期：278例，M-Ⅳ期：141例。本组病例行支气管肺动脉袖状成形肺叶切除256例；肺叶切除联合部分左心房切除重建41例；支气管肺动脉袖状成形联合上腔静脉切除重建90例；肺切除联合部分膈肌切除重建3例；支气管肺动脉袖状成形肺叶切除联合部分左心房切除重建30例；支气管肺动脉袖状成形联合主动脉鞘膜切除10例；右全肺切除联合左心房、右侧全部膈肌、下腔静脉和肝右静脉切除重建1例；肺切除联合气管隆突切除重建10例；支气管肺动脉袖状成形联合气管隆突切除重建和上腔静脉切除重建，或联合上腔静脉和左心房、或联合气管隆突和左心房切除重建55例。手术死亡5例，死亡率为0.97%。516例肺癌病人中141例外周血检测到CK19 mRNA表达，阳性率为27.3%。肺癌病人外周血微转移阳性率与肺癌组织学类型、P-TNM分期、N分期等均有密切关系（*P* < 0.05），但与患者年龄、性别、是否吸烟、原发肿瘤大小、肿瘤部位等均无明显关系（*P* > 0.05）。本组肺癌病人术后中位生存时间为43.74±7.21个月，1年生存率为89.1%，3年生存率为39.3%，5年生存率为19.8%，10年生存率为10.4%。术后生存率与外周血"微转移"、肺癌组织学类型、原发肿瘤大小和淋巴结转移有密切关系（*P* < 0.05）。*Cox*比例风险模型显示"个体化"分子P-TNM分期、外周血"微转移"、病理类型和N分期是预测局部肺癌预后的独立因素。

**结论:**

（1）肺癌病人外周血中存在用常规方法不能检测得到的"微转移"；（2）检测肺癌病人外周血中CK19 mRNA表达应用于肺癌微转移的"分子诊断"和"分子分期，有助于指导选择外科手术适应症、术前新辅助化疗和术后辅助治疗的受益者；（3）基于"分子分期"的局部晚期肺癌的"个体化外科治疗"能明显改善病人的预后，提高治愈率和长期生存率。

## Abstract (en)

**Background and objective:**

Approximately 35%-40% of patients with newly diagnosed non-small cell Lung cancer have locally advanced disease. The average survival time of these patients only have 6-8 months with chemotherapy. The aim of this study is to explore and summarize the probability of detection of micrometastasis in peripheral blood for molecular staging, and for selection of indication of surgical treatment, and beneficiary of neoadjuvant chemotherapy and postoperative adjuvant therapy in locally advanced lung cancer; to summarize the long-time survival result of personalized surgical treatment of 516 patients with locally advanced non-small cell lung cancer based on molecular staging methods.

**Methods:**

CK19 mRNA expression of peripheral blood samples was detected in 516 lung cancer patients by RT-PCR before operation for molecular diagnosis of micrometastasis, personalized molecular staging, and for selection of indication of surgical treatment and the beneficiary of neoadjuvant chemotherapy and postoperative adjuvant therapy in patients with locally advanced nonsmall cell lung cancer invaded heart, great vessels or both. The long-term survival result of personalized surgical treatment was retrospectively analyzed in 516 patients with locally advanced non-small cell lung cancer based on molecular staging methods.

**Results:**

There were 322 patients with squamous cell carcinoma and 194 cases with adenocarcinoma in the series of 516 patients with locally advanced lung cancer involved heart, great vessels or both. There were 112 patients with ⅢA disease and 404 cases with ⅢB disease according to P-TNM staging. There were 97 patients with M-ⅢA disease, 278 cases with M-ⅢB disease and 141 cases with Ⅲ disease according to our personalized molecular staging. Of the 516 patients, bronchoplastic procedures and pulmonary artery reconstruction was carried out in 256 cases; lobectomy combined with resection and reconstruction of partial left atrium was performed in 41 cases; Double sleeve lobectomy combined with resection and reconstruction of super vena cava was carried out in 90 cases; Lobectomy combined with resection and reconstruction of diaphragm was performed in 3 cases; Double sleeve lobectomy combined with resection and reconstruction of partial left atrium was performed in 30 cases; Bronchoplastic procedures and pulmonary artery reconstruction combined with reconstruction of aorta sheath was carried out in 10 cases; Right pneumonectomy combined with resection and reconstruction partial left atrium, total right diaphragm with Dacron, and post cava and right liver vein was performed in one case; Lobectomy combined with resection and reconstruction of carina was carried out in 10 cases; Bronchoplastic procedures and pulmonary artery reconstruction combined with resection and reconstruction of carina and superior vane cava, or combined with superior vena cava and left atrium, or with carina and left atrium was performed in 55 cases in this series. Five patients died of operative complications and the operative mortality was 0.97%. CK19 mRNA expression was found in 141 patients. The positive rate of CK19 mRNA expression was 27.3% in peripheral blood samples in the 516 cases. The positive rates of micrometastasis in peripheral blood was significantly related to histological classification, P-TNM staging and N staging of the cancer (*P* < 0.05), but not to age, sex, smoking status of the patients, and size of primary tumor, and locations of the tumor (*P* > 0.05). The median survival time was 43.74 months. The 1, 3, 5 and 10 year survival rates of the 516 cases was 89.1%, 39.3%, 19.8% and 10.4%, respectively. The postoperative survival rate was remarkably correlated with micrometastasis in peripheral blood, histological classification of the tumor, size of primary cancer and lymph mode involvement (*P* < 0.05). The results of multivariable *Cox* model analysis showed that "personalized molecular P-TNM staging", micrometastasis in peripheral blood, pathological types of the tumor and mediastinal lymph node metastasis of the cancer were the most significant factors for predicting prognosis in the patients with locally advanced nonsmall lung cancer.

**Conclusion:**

(1) Micrometastasis was existed in peripheral blood of patients with lung cancer, which can not be detected with conventional methods. (2) Detecting of CK19 mRNA expression in peripheral blood in lung cancer patients can be used for diagnosis of micrometastasis of lung cancer and "molecular staging" and "molecular P-TNM staging" for lung cancer patients. It will be helpful for selection of surgical treatment indication, the beneficiary of neoadjuvant chemotherapy and postopertive adjuvant therapy in the patients with locally advanced non-small cell lung cancer. (3) Personalized surgical treatment can significantly improve prognosis and increase curative rate and long-term survival rate of locally advanced nonsmall cell lung cancer based on personalized molecular staging.

肺癌是发病率和死亡率增长最快，对人类健康和生命威胁最大的恶性肿瘤之一^[1-20]^。迄今，外科手术仍然是治疗肺癌的首选方法^[21-40]^。早期肺癌手术后的5年生存率可以达到85%左右，即使是Ⅲ期肺癌，外科手术后5年生存率也可以达到20%-41%^[41-55]^。然而，在临床上Ⅰ期肺癌外科手术后复发率可达30%左右^[56-80]^。相反，一些局部晚期肺癌，甚至是侵犯心脏大血管的ⅢB期局部晚期非小细胞肺癌，虽然局部病变较晚，但许多病人并无远处转移，如果能施行肺癌合并受侵组织器官的完全性切除术，仍有30%的患者能获得长期生存^[41-47, 52, 53]^。因此，除了应用临床常用肺癌分期方法对肺癌进行临床分期，选择外科手术适应症外，如何应用分子生物学方法，即"分子分期"，指导肺癌外科手术适应症的选择，以及术前新辅助化疗和术后辅助治疗，是近年来肺癌领域的前沿和重点研究课题^[56-71]^。我们从20世纪90年代初将检测肺癌病人外周血、骨髓和纵隔淋巴结CEA、CK19、Muc-1等基因mRNA表达来诊断肺癌微转移，用于肺癌"分子分期"，指导选择外科手术适应症和术后辅助治疗，进行前瞻性研究^[42-47, 56-64]^。现将我们从1999年到2003年应用"个体化分子分期"外科治疗516例局部晚期肺癌长期生存结果报如下，并对"分子分期"与P-TNM分期等影响预后的因素加以讨论。

## 1 材料与方法

### 1.1 一般资料

肺癌组男性341例，女性175例；年龄24岁-81岁，平均57.86±14.36岁。其中鳞癌322例，腺癌186例；大细胞肺癌4例，腺鳞癌5例，小细胞肺癌2例。P-TNM分期，ⅢA期: 112例，ⅢB期: 404例。"分子分期" (M-TNM分期)：ⅢA期：97例，ⅢB期：278例；Ⅳ期：141例(不管患者P-TNM分期为ⅢA期，还是ⅢB期，只要外周血检测到微转移，则他的M-TNM分期为Ⅳ期)。肺部良性病变组82例，男性52例，女性30例；年龄25岁-69岁，平均52.19±11.36岁。其中支气管扩张29例，炎性假瘤25例，肺结核瘤12例，其他良性病变16例。

### 1.2 肺癌局部侵犯心脏大血管情况

肺癌侵犯仅胸主动脉10例，侵犯上腔静脉90例，侵犯左心房71例，侵犯肺动脉干256例；侵犯膈肌3例，侵犯隆突10例，肺癌同时侵犯2个以上器官76例。

### 1.3 手术术式

(1) 支气管肺动脉袖状成形肺叶切除术256例；(2)肺叶切除合并部分左心房切除重建41例；(3)支气管肺动脉袖状成形肺叶切除合并上腔静脉切除、人造血管重建术90例；(4)肺叶切除合并部分膈肌切除重建术3例；(5)支气管肺动脉袖状成形肺叶切除合并部分左心房切除重建术30例；(6)支气管肺动脉袖状袖状成形肺叶切除合并部分主动脉弓鞘膜切除术10例；(7)右全肺切除合并部分左心房切除重建、右膈肌全切除人工材料重建、下腔静脉和肝右静脉切除人工血管重建术1例；(8)肺切除合并气管隆突切除重建10例；(9)支气管肺动脉袖状成形肺叶切除联合隆突切除重建、上腔静脉切除人工血管重建，或支气管肺动脉袖状成形肺叶切除联合上腔静脉切除人工血管重建，部分左心房切除重建，或支气管肺动脉袖状成形肺叶切除联合隆突切除重建、部分左心房切除重建术55例。

### 1.4 手术方法

所有病人均采用气官插双腔管+静脉麻醉方法，全部病人均行系统性淋巴结"整块"清扫术。

#### 1.4.1 肺切除联合上腔静脉切除重建术

全部病人均经右颈内静脉穿刺，置入Edward 7F或8F的静脉血管鞘导管和5F双腔中心静脉导管，侧孔连接含肝素的生理盐水。5F双腔管备作静脉测压和上腔静脉阻断后持续滴入肝素生理盐水用。右或左股静脉穿刺置入7F或8F的Edward静脉血管鞘导管，用内径3 mm的Edward单向静脉连接管将颈内静脉导管与股静脉导管进行连接，开通三通开关，进行右颈内静脉-股静脉转流。常规右后外侧剖胸切口开胸，探查肺癌侵犯上腔静脉及其它器官情况。解剖左右无名静脉，分别绕过阻断带。切开心包，解剖心包内段上腔静脉，绕过阻断带。解剖奇静脉，分别在近、远心端结扎，缝扎，然后切断奇静脉弓。开放右颈内静脉-股静脉血液转流，分别阻断左右无名静脉和心包内段上腔静脉，持续监测右无名静脉内压力，保持动脉收缩压与右颈内静脉压差 > 50 mmHg(动脉收缩压与右颈内静脉压差=动脉收缩压-右颈内静脉压)。分别在左右无名静脉汇合处下方(或分别切断左右无名静脉)，上腔静脉与右心房汇合处上方切断上腔静脉。用肝素生理盐水冲洗上腔静脉近(或左右无名静脉)、远端血管腔，选择恰当的Gortex带环人造血管行上腔静脉-上腔静脉，或右无名静脉-上腔静脉、左无名静脉-右心房，或左右无名静脉-右心房重建术。用4-0 Prolene带针线，或Gortex-CV3带针线，将人造血管远心端与上腔静脉或左右无名静脉行端端连续缝合。将人造血管修剪成适当的长度后，将人造血管近心端与心包内段上腔静脉或右心房行端端吻合，或人造血管近心端与右心房行端侧吻合，在吻合最后2针前，先开放上腔静脉或左右无名静脉远端阻断带，排除人造血管内的空气，同时，经中心静脉漂浮导管向上腔静脉内持续快速滴注肝素生理盐水。待近心端人造血管吻合、排气完成后，开放近心端上腔静脉阻断钳，或右心房壁上的心耳钳。去除所有阻断带，将漂浮导管经人造血管、右心房、右心室置入肺动脉远端，备作术后监测肺毛嵌压用。根据肿瘤病变情况，施行相应的肺切除术。作系统性"整块"淋巴结清扫术。抗癌药生理盐水冲洗心包腔和胸膜腔，电凝心包切缘。如肺癌侵入上腔静脉或/和右心房腔内，则需在体外循环下施术，摘除右心房内的癌栓。如肺癌侵犯左右无名静脉近心端，则需分别切除左右无名静脉近心端，并用2根人造血管重建左右无名静脉到右心房的血流通道(本组有21例患者采用此术式)。

#### 1.4.2 肺切除联合部分左心房切除重建术

常规剖胸探查，明确肺癌侵犯左心房及其它器官的情况。距肿瘤边缘2 cm-3 cm环形切除心包。距肿瘤边缘3 cm放置1-2把心耳钳，钳夹左心房壁。在心耳钳远心侧1.0 cm处离断左心房壁。用生理盐水反复冲洗左心房腔和切缘，用3-0的Prolene带针线连续交叉缝合左心房切缘两次，然后松开心耳钳，最后用纱布压迫心房壁使缝线针孔渗血自止。根据肺癌情况不同，施行不同术式的肺切除术。清扫各组淋巴结，电凝心包切缘。用抗癌药生理盐水反复冲洗心包腔和胸膜腔。如左心房切除范围过大，需用人造材料缝合修补重建左心房，以扩大左心房腔容积。如肺癌侵犯左心房的同时，伴有左心房腔内癌栓形成，则需借助体外循环施术。手术中需要严格控制静脉液体，尤其是晶体液的输入量和单位时间内的输入速度。

#### 1.4.3 单纯支气管肺动脉袖状成形肺切除术

常规后外侧开胸手术切口入胸探查，明确肺癌侵犯范围和需施行的术式。切开纵膈胸膜，解剖游离左或右肺斜裂，游离出下叶或中间段肺动脉干，绕过阻断带，心包内或心包外解剖游离左或右肺动脉干，绕过阻断带并阻断。解剖游离左或右肺上或肺下静脉干，分别结扎、切断。解剖游离左或右主支气管、上叶或下叶支气管，并分别切除，移去病肺，用3-0带针线行支气管端端间断缝合，吻合术支气管。吻合完成后麻醉师膨肺，确认吻合口无漏气后，用带蒂心包或胸膜包绕支气管吻合口。用生理盐水冲洗肺动脉干近远端血管腔，用4-0 Prolene带针线连续缝合，行肺动脉缝合重建肺动脉。在缝合最后2针前，开放近端肺动脉阻断钳，排除血管腔内的空气。最后开放远端肺动脉阻断钳。如肺动脉吻合口处针孔渗血，可用纱布压迫片刻后可自止。如肺癌侵犯下叶肺动脉达下叶肺动脉起始部或/和下叶背段肺动脉支，可在下叶肺内解剖下叶基底动脉干和背段动脉支，结扎切断背段肺动脉支，将下叶基底动脉干在心包内与左或右肺动脉干行端端吻合术。本组共有24例行下叶基底动脉干与肺动脉干近端的端端吻合术。如肺癌向近端侵犯，进入心包达到心包内肺动脉干时，则需切开心包，在心包内解剖、游离左或右肺动脉干(左肺则常需同时切断动脉导管韧带)，在心包内阻断并切断左或右肺动脉干，然后将左或右肺下叶肺动脉/或基底动脉干在心包内与左或右肺动脉总干行端端吻合术。清扫各组淋巴结，抗癌药生理盐水冲洗胸膜腔或/和心包腔。如支气管，尤其是肺动脉吻合重建时，切断肺下韧带后仍有张力，可在肺下静脉处环形切除部分心包，使肺下静脉游离上提，以减少或消除肺动脉重建的张力。

#### 1.4.4 支气管肺动脉袖状成形肺叶切除合并上腔静脉切除人造血管置换术

右肺中心型肺癌同时侵犯肺动脉干和上腔静脉时，对这样的患者可以有条件和有选择地施行支气管肺动脉袖状成形肺叶切除合并上腔静脉切除、人造血管重建术。全部病人均经右颈内静脉穿刺，置入Edward 7F或8F的静脉血管鞘导管和5F双腔中心静脉导管，侧孔连接含肝素的生理盐水。5F双腔管备作静脉测压和上腔静脉阻断后持续滴入肝素生理盐水用。右或左股静脉穿刺置入7F或8F的Edward静脉血管鞘导管，用内径3 mm的Edward单向静脉连接管将颈内静脉导管与股静脉导管进行连接，开通三通开关，进行右颈内静脉-股静脉转流。常规右后外侧剖胸切口开胸，探查肺癌侵犯上腔静脉及右肺动脉总干的情况。首先按1.4.1的方法施行上腔静脉切除、人造血管置换术，然后按1.4.3的方法施行支气管肺动脉袖状成形肺叶切除术。本组有90例患者实施了支气管肺动脉袖状成形肺叶切除合并上腔静脉切除人造血管置换术(图 1)。

**1:**
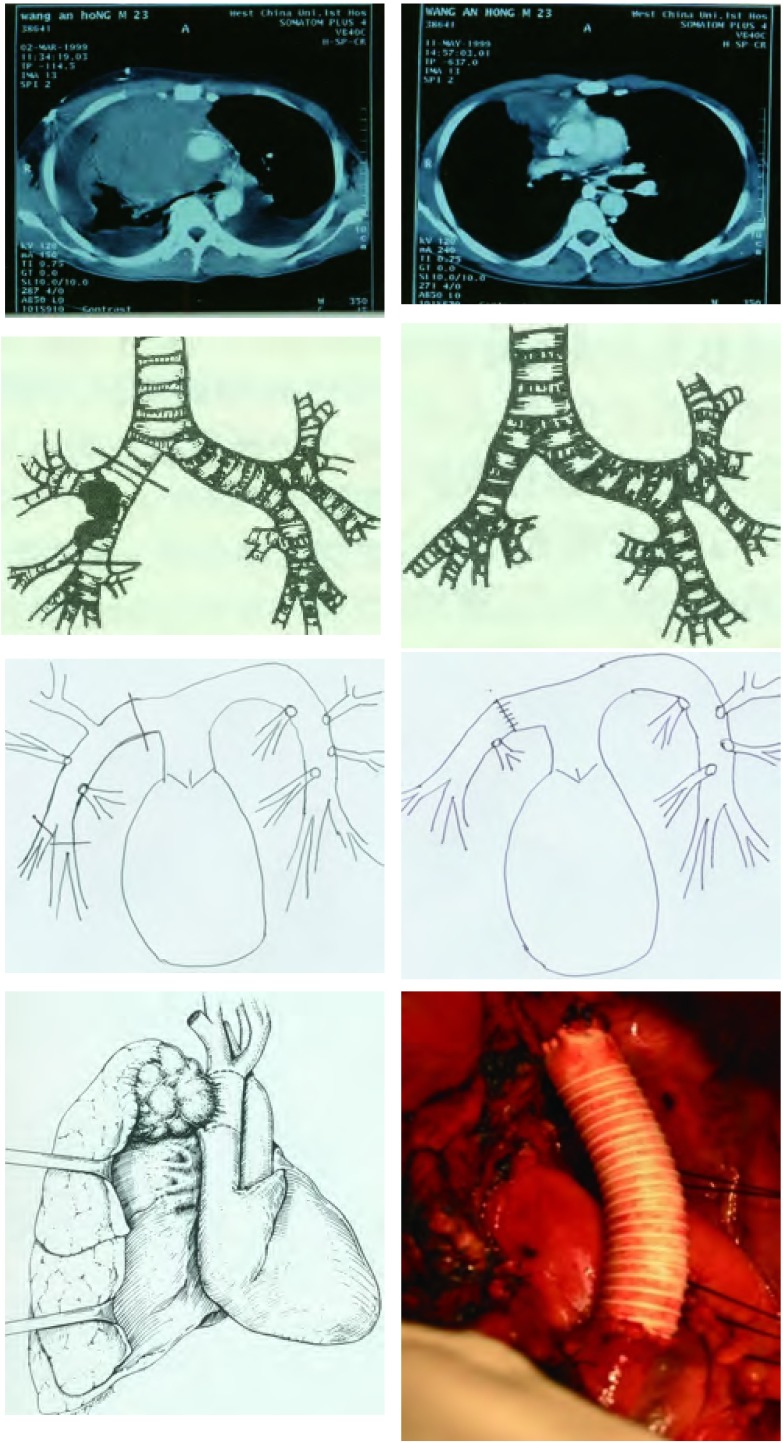
男性，29岁。右肺中心型肺鳞癌侵犯气管隆突、右肺动脉总干和上腔静脉。术前给予GP方案化疗2周期。新辅助化疗前后外周血CK19 mRNA检测均阴性。施行支气管肺动脉袖状成型右肺中上叶切除，气管隆突切除重建，上腔静脉切除Gortex人造血管重建，病人术后存活已14年，无肿瘤复发转移。 Male, 29 year old. Right central type of squamous cell carcinoma of the lung. The tumor invaded trachea, carina, SVC and RMPA. 2 cycles of GP regimen was given before operation. Detection of micrometastasis were negative both before and after neoadjuvast chemotherapy. C-TNM and M-TNM(CK19 detection was negative) staging was ⅢB. Double sleeve right upper-middle lobectomy, carina resection and reconstruction and SVC graft were performed. The patient has survived over 14 years without recurrence of the cancer after operation.

#### 1.4.5 支气管肺动脉袖状成形肺叶切除联合隆突切除重建、上腔静脉切除人工血管重建术

右肺上叶中心型肺癌同时侵犯隆突、右肺动脉干和上腔静脉时，对这样的患者可以有条件和有选择地施行支气管肺动脉袖状成形肺叶切除合并气管隆突切除重建、上腔静脉切除人造血管重建术。全部病人均经右颈内静脉穿刺，置入7F或8F的Edward静脉血管鞘导管和5F双腔中心静脉导管，侧孔连接含肝素的生理盐水。5F双腔管备作静脉测压和上腔静脉阻断后持续滴入肝素生理盐水用。右或左股静脉穿刺置入7F或8F的Edward静脉血管鞘导管，用内径3 mm的Edward单向静脉连接管将颈内静脉导管与股静脉导管进行连接，开通三通开关，进行右颈内静脉-股静脉转流。常规右后外侧剖胸切口开胸，探查肺癌侵犯上腔静脉及右肺动脉总干的情况。首先按1.4.1的方法施行上腔静脉切除人造血管置换术，然后游离气管下段、左主支气管、右中间干支气管/或右下叶支气管/或右基底干支气管，分别切断气管下段、左主支气管、右中间段支气管/或右下叶支气管/或右基底干支气管，用3-0带针线将左主支气管-右中间段支气管(或右下叶支气管/或右基底干支气管)与气管下段行端-端吻合重建气管支气管。吻合结束后膨肺试水检查吻合口是否漏气。最后按1.4.3的方法施行右肺动脉干袖状成形重建术。本组共有35例患者施行支气管肺动脉袖状成形肺叶切除联合隆突切除重建、上腔静脉切除人工血管重建术(图 2、图 3)。

**2:**
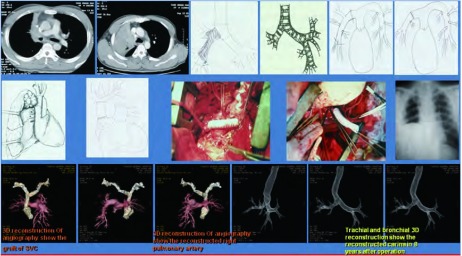
男性，55岁。右肺中心型肺鳞癌侵犯气管隆突、右肺动脉总干和上腔静脉，外周血CK19 mRNA检测阴性。施行支气管肺动脉袖状成形右肺中上叶切除、气管隆突切除重建，上腔静脉切除Gortex人造血管重建，病人术后存活已10年，无肿瘤复发转移。 Male, 55 year old. Right central type of squamous cell carcinoma of the lung. The tumor invaded trachea, carina, SVC and RMPA. Detection of micrometastasis was negative. Both C-TNM and M-TNM staging was ⅢB. Double sleeve right upper-middle lobectomy, carina resection and reconstruction and SVC graft were performed. The patient has survived over 10 years without recurrence of the cancer after operation.

**3:**
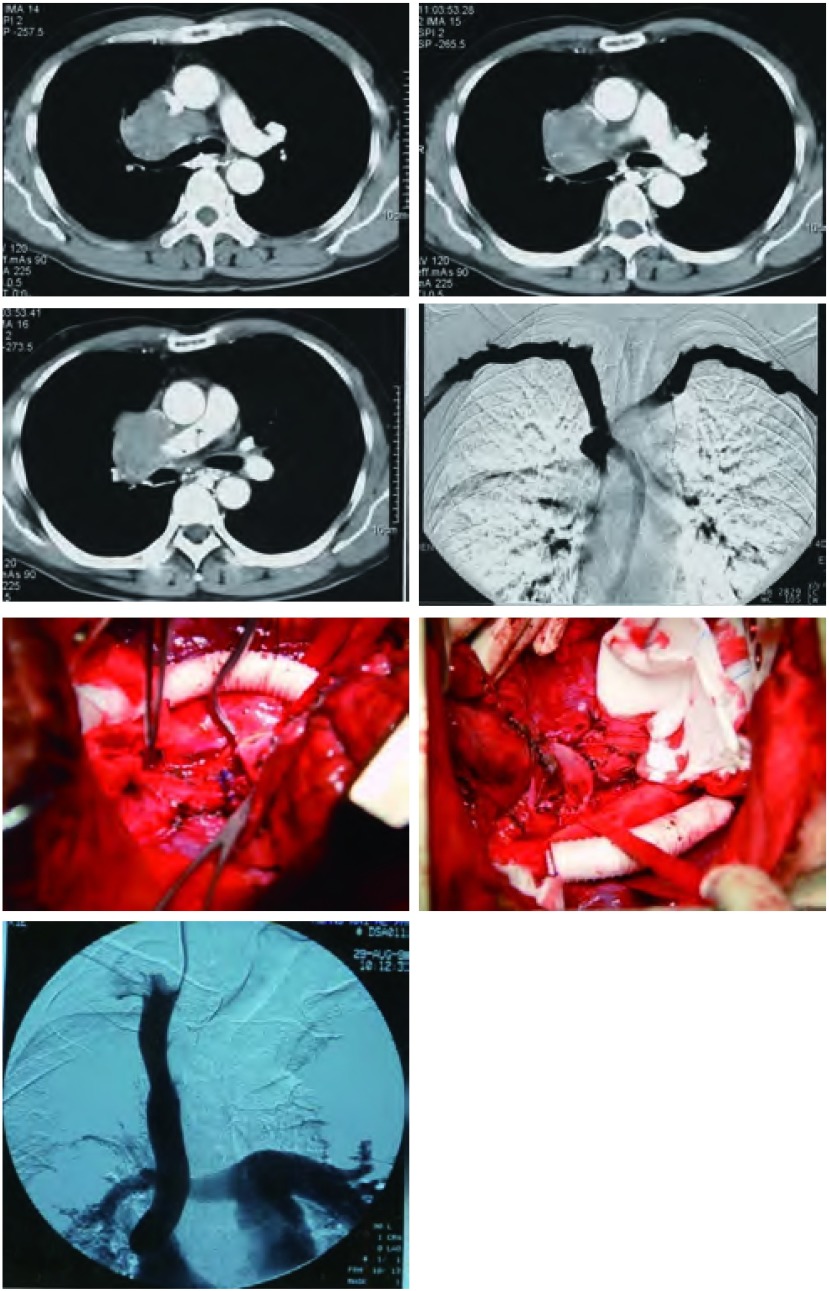
男性，49岁。右肺中心型小细胞肺癌侵犯气管隆突、右肺动脉总干和上腔静脉，外周血CK19 mRNA检测阴性。施行支气管肺动脉袖状成形、右肺中上叶切除，气管隆突切除重建，上腔静脉切除Gortex人造血管重建，病人术后存活已10年，无肿瘤复发转移。 Male, 49 year old. Right central type of small cell lung cancer invaded trachea, carina, SVC and RMPA. C-TNM and M-TNM staging was ⅢB. Double sleeve right upper-middle lobectomy, carina resection and reconstruction and SVC graft were performed. The patient has survived over 7 years without recurrence of the cancer after operation.

#### 1.4.6 支气管肺动脉袖状成形肺叶切除联合上腔静脉切除人工血管重建、部分左心房切除重建术

右肺上叶中心型肺癌可以同时侵犯右肺动脉干、上腔静脉和左心房，对这样的患者可以有条件和有选择地施行支气管肺动脉袖状成形肺叶切除联合上腔静脉切除人工血管重建、部分左心房切除重建术。全部病人均经右颈内静脉穿刺，置入7F或8F的Edward静脉血管鞘导管和5F双腔中心静脉导管，侧孔连接含肝素的生理盐水。5F双腔管备作静脉测压和上腔静脉阻断后持续滴入肝素生理盐水用。右或左股静脉穿刺置入7F或8F的Edward静脉血管鞘导管，用内径3 mm的Edward单向静脉连接管将颈内静脉导管与股静脉导管进行连接，开通三通开关，进行右颈内静脉-股静脉转流。常规右后外侧剖胸切口开胸，探查肺癌侵犯上腔静脉及右肺动脉总干的情况。首先按1.4.1的方法施行上腔静脉切除人造血管置换术，然后按1.4.2的方法施行部分左心房切除重建术，最后按1.4.3的方法施行支气管肺动脉干袖状成形右肺上叶或右肺中上叶切除重建术。本组共有10例患者施行支气管肺动脉袖状成形肺叶切除联合上腔静脉切除人工血管重建、部分左心房切除重建术。

#### 1.4.7 支气管肺动脉袖状成形肺叶切除联合隆突切除重建、部分左心房切除重建术

右肺上叶中心型肺癌可以同时侵犯气管隆突、右肺动脉总干和左心房。对这样的患者可以有条件和有选择地施行支气管肺动脉袖状成形肺叶切除联合隆突切除重建、部分左心房切除重建术。常规右后外侧剖胸切口开胸，探查肺癌侵犯气管隆突、右肺动脉总干和左心房的情况。首先，按照1.4.2的方法施行部分左心房切除重建术，然后游离气管下段、左主支气管、右中间干支气管/或右下叶支气管/或右基底干支气管，分别切断气管下段、左主支气管、右中间段支气管/或右下叶支气管/或右基底干支气管，用3-0带针线将左主支气管-右中间段支气管(或右下叶支气管/或右基底干支气管)与气管下段行端-端吻合重建气管支气管。吻合结束后膨肺试水检查吻合口是否漏气。最后，按照1.4.3的方法施行支气管肺动脉干袖状成形右肺上叶或右肺中上叶切除重建术。本组共有10例患者施行支气管肺动脉袖状成形肺叶切除联合隆突切除重建、部分左心房切除重建术。

#### 1.4.8 右全肺切除合并部分左心房切除、右全膈肌切除人工材料重建、下腔静脉肝右静脉切除重建术

本组有1例女性患者，在外地医院因右肺癌在一家三甲医院开胸手术。术中发现肺癌侵犯左心房、右膈肌和胸腔内段下腔静脉，而仅行肺癌肿瘤活检。剖胸探查术后，病理报告为小细胞肺癌。术后用EP方案化疗7个周期，化疗期间加胸部放疗65 Gy。放疗和化疗后肿瘤不但未缩小，相反，较治疗前明显长大。患者要求手术治疗而来我院就诊。在充分术前准备后，在全麻下施行右全肺切除联合部分左心房切除重建，右全膈肌切除人工材料重建，下腔静脉肝右静脉切除人工血管重建术。首先，在右侧腹股沟解剖出股动脉和股静脉，分别作动脉和静脉插管，连接体外循环机。作标准右后外开胸切口入胸，游离胸膜粘连。切开心包，游离、结扎、切断右肺动脉总干。用心耳钳钳夹左心房，并切断。然后用3-0 Prolene带针线连续缝合左心房。游离右主支气管，距隆突约0.5 cm切断右主支气管，用3-0带针线间断缝合右主支气管残段，膨肺无漏气后，用心包片包埋支气管残段。从右侧膈肌附着处游离、切断膈肌，显露腹腔和腹腔内段下腔静脉，绕过阻断带；游离心包内段下腔静脉，绕过阻断带。体内肝素化后进行下半身体外循环，腹腔内阻断下腔静脉远端，心包内阻断下腔静脉近端。分别切断下腔静脉远心端和近心端，切断肝右静脉近心端。将右全肺，连同受侵的部分心包、左心房、右侧膈肌，以及下腔静脉和一段肝右静脉。分别用4-0 Prolene线将带环的Gortex人造血管与下腔静脉远端和近心端行端-端吻合，最后2针排气后打结。最后将4块20 cm×20 cm的医用涤纶布缝合成为1块40 cm×40 cm的大块涤纶布，将该块涤纶布重建右膈肌(图 4)。

**4:**
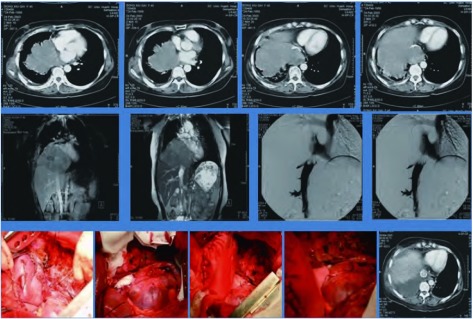
女性，48岁。右肺中心型小细胞肺癌侵犯左心房、下腔静脉、肝右静脉和右膈肌。于2002年5月行剖胸探查活检术，病理报告为小细胞肺癌。术后给予EP方案化疗7周期，中间放疗65 Gy，肿瘤进展。所有临床检测无远处转移，外周血检测CK19阴性。于2003年1月在股动-静脉转流下，施行右全肺切除联合部分左心房、右膈肌、下腔静脉、肝右静脉切除人工血管重建。术后7年无肿瘤复发转移。 Female, 48 year old. Right central type of small cell lung cancer invaded left atrium, postcava, right liver vein and right diagram. C-TNM and M-TNM staging was ⅢB. Right exploratory thoracotomy and biopcy was performed in May, 2002. After exploratory thoracotomy. 7 cycles of chemotherapy with EP regimen and 65 Gy radiotherapy were given from June to December of 2002. Under CBP at right femoral artery and vein, right pneumonectomy, partial resection and reconstruction, resection and reconstruction of right diagram with dacron, resection and reconstruction of right liver vein, and postcava graft were performed in Jan, 2003. The patient has survived over 7 years without recurrence of the cancer after operation.

### 1.5 术前新辅助化疗

本组对115例病人进行了术前新辅助化疗。化疗方案主要NP和GP方案，均给2周期新辅助化疗。新辅助化疗前后均行外周血"微转移"检测。新辅助化疗后疾病缓解或/和稳定、且新辅助化疗前后"微转移"检测均为阴性者则给予外科手术治疗，否则只能继续化疗和加放疗治疗。

### 1.6 围手术期处理

对于侵犯心脏大血管的局部晚期肺癌，施行肺切除合并受侵心脏、大血管切除重建术的患者，围术期处理十分重要。由于手术创伤大，手术时间较长，手术后患者易出现心肺并发症，尤其是较长时间阻断一侧肺动脉干和伴上腔静脉综合征的病人，很容易并发肺再灌注损伤发生严重的肺换气功能异常和心功能不全，因此，这类病人围手术期监护及处理十分重要。所有患者都应进入重症监护病房监护治疗2 d-3 d，并应重点做好下列监护和处理：(1)加强呼吸和血流动力学的监测，密切注意呼吸和循环的变化。(2)每一个患者都应置CVP和漂浮导管，以监测和记录CVP、肺毛嵌压、心输出量及心脏指数。(3)控制静脉输液量和输液速度：术中离断左心房后立即限制静脉输液速度和24 h输液量。(4)术后1周内，每天体液负平衡500 mL-1, 000 mL左右，即当天的静脉输液量=前一天的尿量+前一天的额外丢失量(如胸腔引流液)-(500 mL-1, 000 mL)。此外，每天的计划输液量还应24 h均匀缓慢输入。(5)术后呼吸机辅助呼吸6 h-12 h(如在体外循环下施术，则延长至12 h-24 h)，并加用PEEP。脱机后再吸氧治疗3 d-5 d。(6)利尿治疗3 d-4 d(一般用速尿20 mg，每天1-2次)。如出现肺水肿、中心静脉压和肺毛嵌压升高者，则应加大速尿的用量，每次20 mg-40 mg，每天3-4次，直到肺水肿治愈。(7)有效广谱抗生素治疗5 d-7 d。(8)加强呼吸道的管理，进行有效的胸部物理治疗，保持呼吸道通畅，防止呼吸道并发症。(9)施行上腔静脉切除重建术，术后前48 h内给予潘生丁或低分子肝素抗凝治疗，拔除胸腔引流管后，则用华法林抗凝治疗，使凝血酶原时间延长1.2倍-1.5倍。

### 1.7 手术前后多学科综合治疗

本组病人大多数为ⅢB期肺癌。在我们早期的病例，多是手术和化疗同期进行，并将其命名为"围术期强化化疗"。其具体实施方法是：手术日麻醉开始，注射抗菌素后，手术开始前给患者静脉推注或/和滴注抗肿瘤药物，主要应用NP方案和GP方案。术后第21天，可开始第2个周期的化疗，化疗2个周期后，加放疗1周期，然后再化疗2-3个周期。化疗的同时需注意免疫功能、肝肾功能和骨髓功能的保护。在化疗期间可选用日达仙作免疫治疗。术后放疗的剂量应与非手术根治性放疗有所不同，放疗前已行化疗者与未化疗者，放疗剂量亦应不同。术后放疗应较非手术根治放疗剂量至少小10 Gy，已作过化疗者应较未作化疗者放疗剂量小10 Gy-15 Gy，以减少术后放疗对肺功能和其它正常胸部器官的放射性损伤。放疗总剂量在45 Gy-55 Gy之间。

此外，我们对部分患者先行术前新辅助化疗2个周期，化疗前后作外周血微转移的检测，术前新辅助化疗前后微转移检测均阴性者，则在化疗结束后3周施行外科手术。

### 1.8 随访

所有病人均有专人进行随访。术后第1-3年，每3-4个月复查一次，每次复查包括胸部、上腹部强化CT，头部强化MRI和肿瘤标志物检测，随访时间7年-11年。计算和统计肿瘤复发转移和病人的生存情况。生存时间从术后1个月到死亡时间和随访截止时间。对失访病人按死亡计算。

### 1.9 外周血微转移检测

#### 1.9.1 血标本的收集

研究对象为非小细胞肺癌(non-small cell lung cancer, NSCLC)患者肿瘤组织、外周血标本，均取自四川大学华西医院胸心外科接受手术治疗的患者，共516例。同期肺良性病变组织患者56例。健康人血液标本来自志愿者。术前抽取研究对象的外周血标本2 mL，以及健康志愿者外周血2 mL，肝素抗凝后经液氮速冻，置于-80 ℃超低温冰箱中保存；组织标本取1. 0 cm^3^左右保存于液氮中备用。

#### 1.9.2 肺癌细胞株

灵敏度实验所需肺癌细胞株A549由美国国立癌症研究所(NCI)提供，由四川大学华西医学中心肿瘤研究所培养。

#### 1.9.3 主要试剂

异硫氰酸胍、焦碳酸二乙酯、醋酸钠、柠檬酸三钠、氯仿等均购自华美生物公司；逆转录酶MMLV购自美国Promega公司；RNA酶抑制剂Rnsin、TaqDNA聚合酶购自成都同正生物公司；DNA Marker (DL2000)购自成都天泰生命科技公司。

#### 1.9.4 引物

引物由上海生工生物工程有限公司合成。参照*CK19*基因序列，设计引物^[81, 82]^。外引物(A和B)分别位于第3和第6外显子，内引物(C和D)分别位于第4和第5外显子，各扩增1, 069 bp和745 bp的片段。A: 5'-AAGCTAACCATGCAGAACCTCAACGACCGC-3'；B: 5' -TTATTGGCAGGTCGGAGAAGAGCC-3'；C: 5'-TCCCGCGACTACAGCCACTACTACACGACC-3'；D: 5'-CGCGACTTGATGTCGATGAGCCGCTGGTAC-3'。

β-actin作为内对照标准，参照其基因序列^[83]^设计引物，产物约为200 bp。

A : 5'-TCATCACCATTGGCA ATGAG- 3'；B: 5'-CACTGTGTTGGCGTACAGGT-3'。

#### 1.9.5 主要仪器

PCR扩增仪2400型(美国PE公司)；低温高速离心机(美国Beckman公司)；-150 ℃超低温冰箱(日本Sanyo公司)；低温高速离心机(美国Beckman公司)；高压蒸汽灭菌设备(日本Sanyo公司)。

#### 1.9.6 方法

##### 1.9.6.1 RNA的提取及纯化

肺癌组织RNA的提取采用AGPC一步法^[84]^。外周血及标本RNA的提取采用改良AGPC一步法^[85]^，纯化采用玻璃乳法^[86]^。

##### 1.9.6.2 CK19巢式RT-PCR

将100 ng RNA加入以下逆转录体系中：5X缓冲液5 μL，逆转录酶400 U，Rnsin 50 U，dNTP(200 mmol/μL)3 μL，下游引物(20 pmol/μL) 1 μL，总体积25 μL，70 ℃水浴5 min后37 ℃孵育1 h。取10 μL逆转录产物加入以下混合物中：10X缓冲液5 μL，dNTP (200 μmol/μL)4 μL，MgCl_2_(25 mmol/μL)3 μL，外引物(20 pmol/μL)各3 μL，Taq聚合酶3 U/2 μL，总体积50 μL。反应条件为：94 ℃、15 min，55 ℃、5 min，加入Taq聚合酶，94 ℃、1 min，55 ℃、11 min，72 ℃、1 min，循环扩增30次，72 ℃终延伸7 min。取3 μL产物加入以下混合物中：10X缓冲液2.5 μL，dNTP(200 μmol/μL) 4 μL，MgCl_2_(25 mmol/μL)2 μL，内引物(20 pmol/μL)各3 μL，Taq聚合酶1.5 U/2 μL，总体积25 μL。反应条件为：94 ℃、5 min，94 ℃、1 min，55 ℃、1 min，72℃、1 min，循环扩增30次，72 ℃终延伸7 min，得到循环产物备用。

##### 1.9.6.3 β-actin RT-PCR

将100 ng RNA加入以下逆转录体系中：10X上样液2.5 μL，dNTP(200 μmol/μL)2 μL，MgCl_2_ (25 mmol/μL)1 μL，引物(20 pmol/μL)各3 μL，Taq聚合酶1.5 U/2 μL，总体积25 μL。反应条件为：94 ℃、5 min，48 ℃、5 min，加入Taq聚合酶，94 ℃、1 min，48 ℃、45 s，72 ℃、45 s，循环扩增30次，72 ℃终延伸7 min，得到循环产物备用。

##### 1.9.6.4 PCR产物鉴定

取10 μL反应产物加入2 μL上样液在1%琼脂糖凝胶中电泳，经EB染色得到特异性条带者为阳性检出。

##### 1.9.6.5 灵敏度实验

将生长良好的A549肺癌细胞株用少量胰蛋白酶消化，加入10 mL 0.9% NS混匀。取一滴于显微镜下用白细胞计数仪计数，得到每毫升悬液所含细胞数。调整液体体积至每毫升悬液含1×10^6^个细胞，依次稀释悬液浓度至1×10^6^/mL、1×10^5^/mL、1×10^4^/mL、1× 10^3^/mL、1×10^2^/mL、1×10/mL和1/mL等7个数量级细胞浓度。提取细胞RNA作CK19 mRNA的RT-PCR分析。

## 2 统计学处理方法

采用SPSS 17.0统计软件处理数据，检验水准确定*P* < 0.05为有显著性差异。应用*Kaplan-Meier*生存曲线分析生存率，应用*Log-rank*分析各组间生存率差异。应用*Cox*比例风险模型分析可能影响肺癌预后的各项因素。应用*χ*^2^检验分析各组间外周血肺癌微转移的阳性率。

## 3 结果

### 3.1 术后并发症及手术死亡率

本组患者术后发生并发症74例次，其中急性肺水肿6例，肺部感染48例，心律紊乱21例，呼吸衰竭7例。2例因肺部感染，2例因呼吸衰竭，1例因深静脉血栓、肺栓塞死亡，死亡率为0.96%。

### 3.2 外周血微转移检出率

#### 3.2.1 外周血标本总RNA的鉴定

采用异硫氰胍-酚-氯仿一步法提取组织及淋巴结RNA；采用改良一步法提取外周血和骨髓RNA，磁珠法进行纯化。经过1%琼脂糖凝胶电泳鉴定，18S和28S条带清晰可见(图 5)。

**5:**
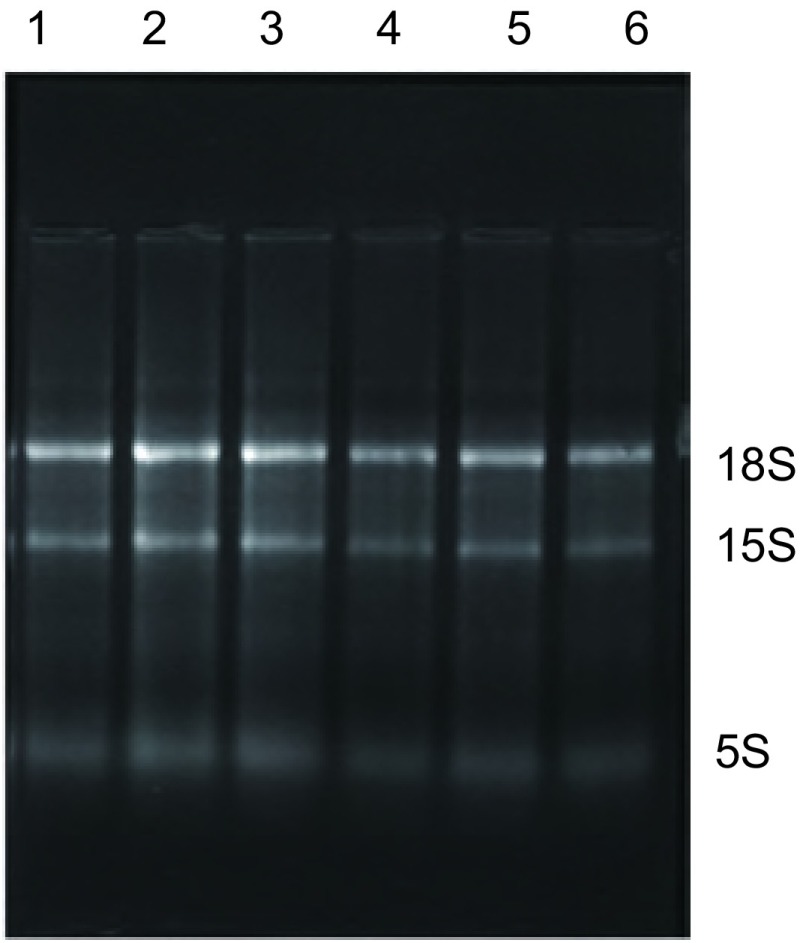
外周血总RNA的1%琼脂凝胶电泳图谱 Agarose electrophoresis of total RNA from peripheral blood sample. 1, 2, 3: Peripheral blood sample from patients with benign pulmonary disease; 4, 5, 6: Peripheral blood sample from patients with lung cancer.

#### 3.2.2 肺癌病人外周血微转移检测结果

##### 3.2.2.1 肺癌组和肺良性病变组肺组织CK19 mRNA表达结果

本组519例肺癌患者肺癌组织标本均检测出*CK19*基因的阳性表达，表达率为100%(516/516)。82例肺良性病变组织标本中，81例扩增出CK19 mRNA的特异性条带，阳性率为98.8%(81/82)；1例结核瘤标本未扩增出特异性条带。肺癌组和肺良性病变组CK19 mRNA表达比较无显著性差异(*P* > 0.05)(表 1)。图 5为8例肺癌病人外周血CK19 mRNA表达电泳图。其中2、3、5、7和9泳道CK19 mRNA表达阳性，而4、6和8泳道CK19 mRNA表达均为阴性。图 6中2、3、5、7和9泳道检测的肺癌病人外周血CK19 mRNA表达均为阳性，但4和6泳道2例NSCLC病人、8泳道肺良性病变病人外周血CK19 mRNA表达均为阴性。516例肺癌病人中141例外周血检测到CK19 mRNA表达，阳性率为27.3%；而82例肺良性病变病人外周血中均未检测到CK19 mRNA表达。两组间阳性率比较具有显著性差异(*P* < 0.05)(表 2)。

**1:** 肺癌组织和肺良性病变肺组织CK19 mRNA表达比较 Comparison of CK19 mRNA expression between lung cancer tissue and benign pulmonary lesion tissue

Groups	*n*	CK19 mRNA		Positive Rate (%)
		+	-	
Lung cancer	516	516	0	100
Benign pulmonary lesion	82	81	1	98.8
*P*				> 0.05

**6:**
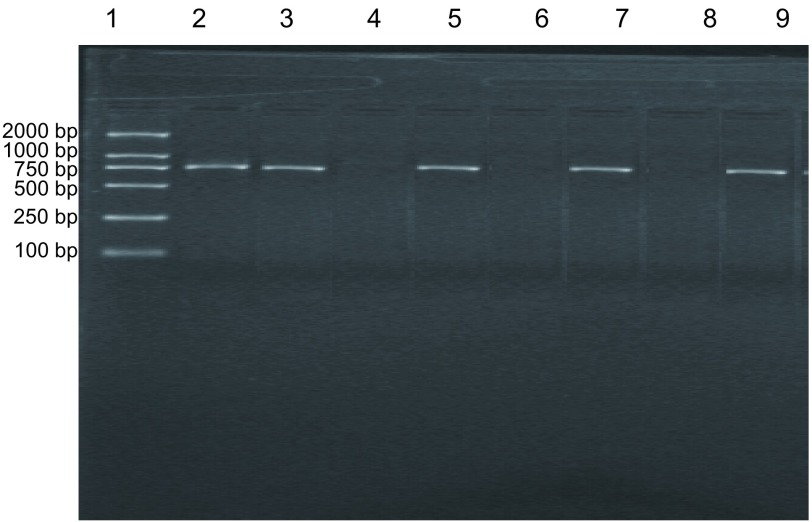
肺癌病人外周血CK19 mRNA表达的RT-PCR分析结果 RT-PCR analysis of CK19 mRNA in peripheral blood samples from patients with lung cancer. 1: DNA marker; 2-9: Peripheral blood samples from lung cancer patients.

**2:** 肺与和肺良性病变病人外周血CK19 mRNA表达比较 Comparison of CK19 mRNA expression in peripheral blood between the patients with lung cancer and benign pulmonary lesion

Groups	*n*	CK19 mRNA		Positive Rate (%)
		+	-	
Lung cancer	516	141	0	27.3
Benign pulmonary lesion	82	0	82	0.0
*P*				< 0.001

**7:**
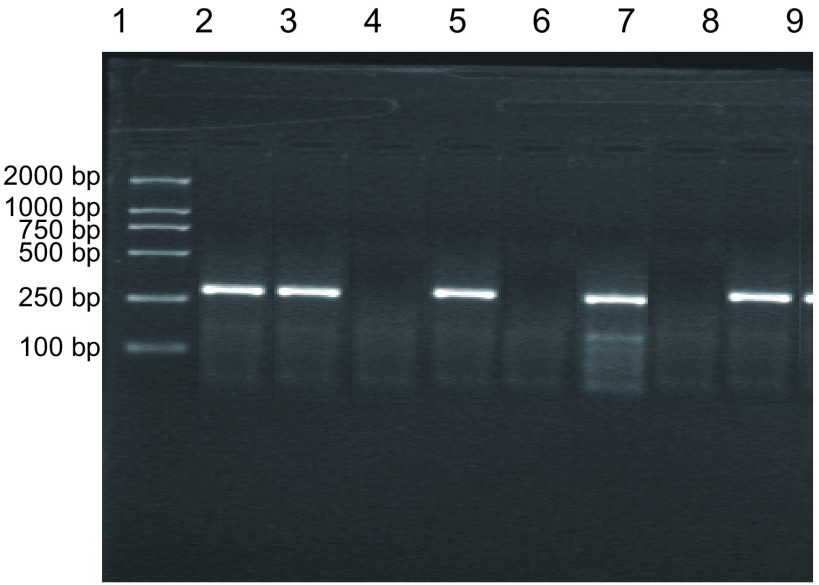
肺癌病人外周血CK19 mRNA表达的RT-PCR分析结果 RT-PCR analysis of CK19 mRNA in peripheral blood samples from patients with lung cancer. 1: DNA marker; 2-4: Peripheral blood samples from lung cancer patients; 5, 7, 9: Peripheral blood samples from lung cancer patients; 6, 8: Peripheral blood samples from patients with benign pulmonary diseases.

#### 3.2.3 肺癌病人外周血微转移与肺癌病例生理特征的关系

肺癌病人外周血微转移阳性率与肺癌组织学类型、P-TNM分期、N分期等均有密切关系(*P* < 0.05)，但与患者年龄、性别、是否吸烟、原发肿瘤大小、肿瘤部位等均无明显关系(*P* > 0.05)(表 3)。

**3:** 肺癌外周血微转移与肺癌临床病理生理特征的相关关系 The relationship between peripheral blood OMs and clinico-pathologic characteristics in patients with lung cancer

Characteristics	*n*	Peripheral blood OMs		*P*
		+	-	
Age				> 0.05
< 50	246	72	174	
> 50	270	69	201	
Sex				> 0.05
Male	341	79	262	
Female	175	62	113	
Smoking history				> 0.05
Yes	340	94	246	
No	176	47	129	
Primary tumor				> 0.05
T3	111	32	79	
T4	405	109	296	
Histologic classification				< 0.001
Sqamous Ca.	332	38	304	
Non-sqamous Ca.	194	103	91	
N staging				< 0.001
N0+N1	288	42	246	
N2	231	99	132	
Stage				< 0.001
ⅢA	112	12	100	
ⅢB	404	129	275	

#### 3.2.4 外周血微转移检测的灵敏度

本研究结果发现CK19 mRNA的RT-PCR分析的灵敏度可以达到10^-6^(10个癌细胞/mL)，而10^-7^级(1个癌细胞/mL)的细胞悬液未检测出CK19的阳性表达(图 8)。

**8:**
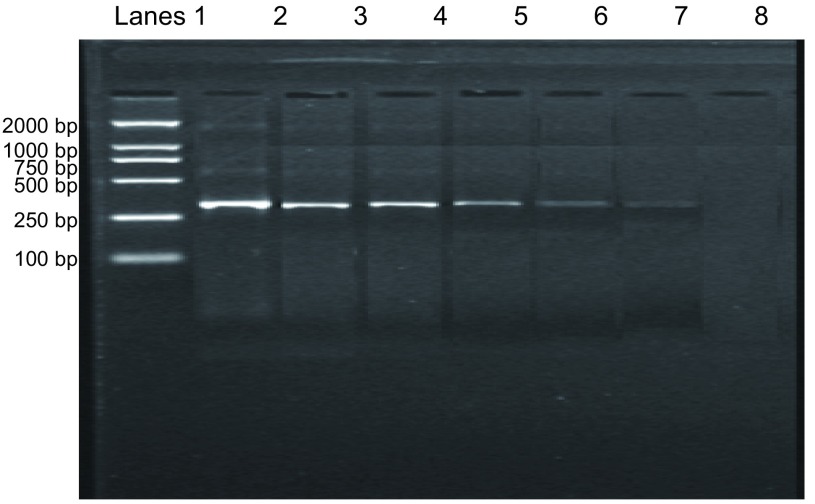
CK19 mRNA表达灵敏度实验 Sensitivity test of the RT-PCR method for detection of CK19 mRNA expression. 1: DNA marker; 2: 1×10^6^/mL; 3: 1×10^5^/mL; 4: 1× 10^4^/mL; 5: 1×10^3^/mL; 6: 1×10^2^/mL; 7: 1×10/mL; 8: 1/mL.

### 3.3 术后生存

本组516例局部晚期肺癌中位生存时间为43.74±7.21个月；1年生存率为89.1%，3年生存率为39.3%，5年生存率为19.8%，10年生存率为10.4%。鳞癌组术后1、3、5和10年生存率分别为93.5%、48.1%、32.6%和10.4%；非鳞癌组术后1、3、5和10年生存率分别为86.8%、20.8%、11.2%和3.8%；鳞癌和非鳞癌组间术后生存率比较有显著性差异(*P* < 0.05)(图 9)。T3肺癌术后1、3、5和10年生存率分别为91.4%、39.2%、27.0%和8.5%；T4肺癌术后1、3、5和10年生存率分别为89.2%、30.4%、15.3%和4.4%；T3与T4肺癌组间术后生存率比较有显著性差异(*P* < 0.05)(图 10)。N1肺癌术后1、3、5、10年生存率分别为92.4%、39.5%、26.4%和14.8%；N2肺癌术后1、3、5和10年生存率分别为89.2%、37.2%、22.1%和2.6%；N1与N2肺癌组间术后生存率比较有显著性差异(*P* < 0.05)(图 11)。ⅢA期(P-TNM分期)肺癌术后1、3、5和10年生存率分别为91.4%、39.1%、27.8%和9.6%；ⅢB期(P-TNM分期)肺癌术后1、3、5和10年生存率分别为89.3%、33.0%、16.1%和5.4%；ⅢA与ⅢB期肺癌组间术后生存率比较有显著性差异(*P* < 0.05)(图 12)。不伴有外周血"微转移"肺癌病人术后1、3、5和10年生存率分别为94.8%、56.6%、38.9%和14.5%；伴有外周血"微转移"肺癌病人术后1、3、5和10年生存率分别为84.6%、9.7%、0.5%和0%；伴"微转移"与不伴"微转移"肺癌组间术后生存率比较有显著性差异(*P* < 0.05)(图 13)。

**9:**
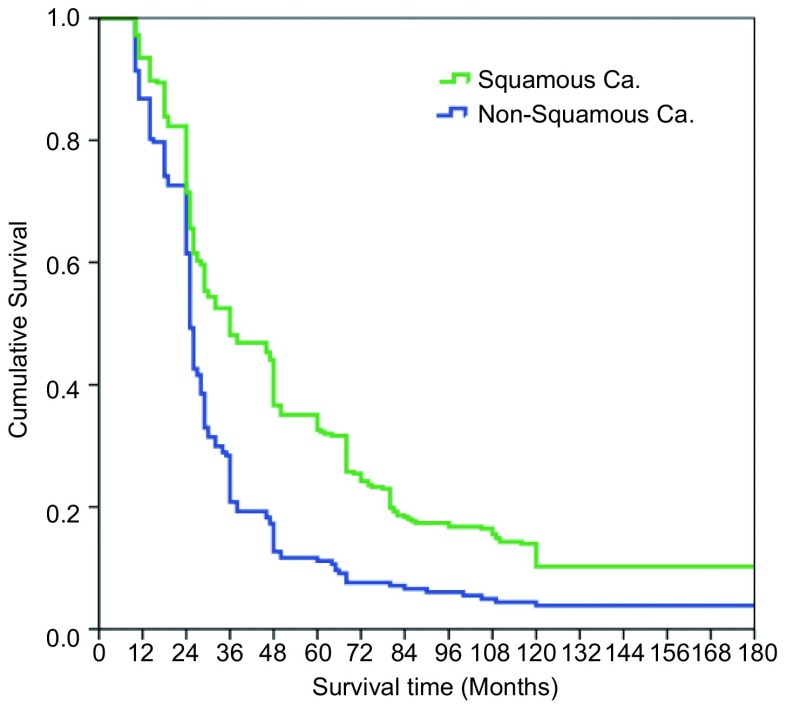
鳞癌组与非鳞癌组术后生存曲线（*Kaplan-Meier*曲线）（对数秩和检验，*P* < 0.05） *Kaplan-Meier* survival curves between the patients with squamous cell carcinoma and nonsquamous cell carcinoma of the lung (*Log-rank* test, *P* < 0.05)

**10:**
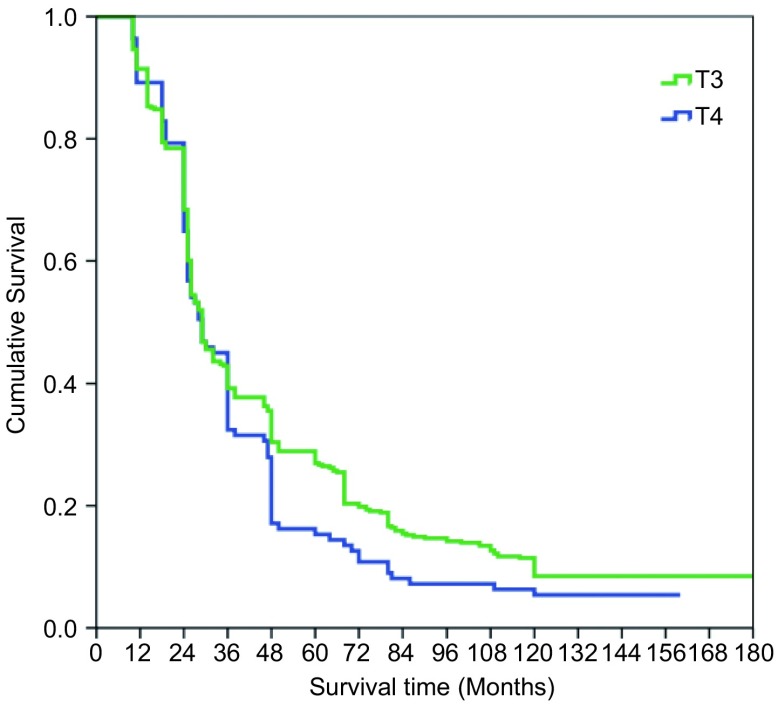
T3与T4肺癌术后生存曲线（*Kaplan-Meier*曲线）（对数秩和检验，*P* < 0.05） *Kaplan-Meier* survival curves between the patients with T3 and T4 disease (*Log-rank* test, *P* < 0.05)

**11:**
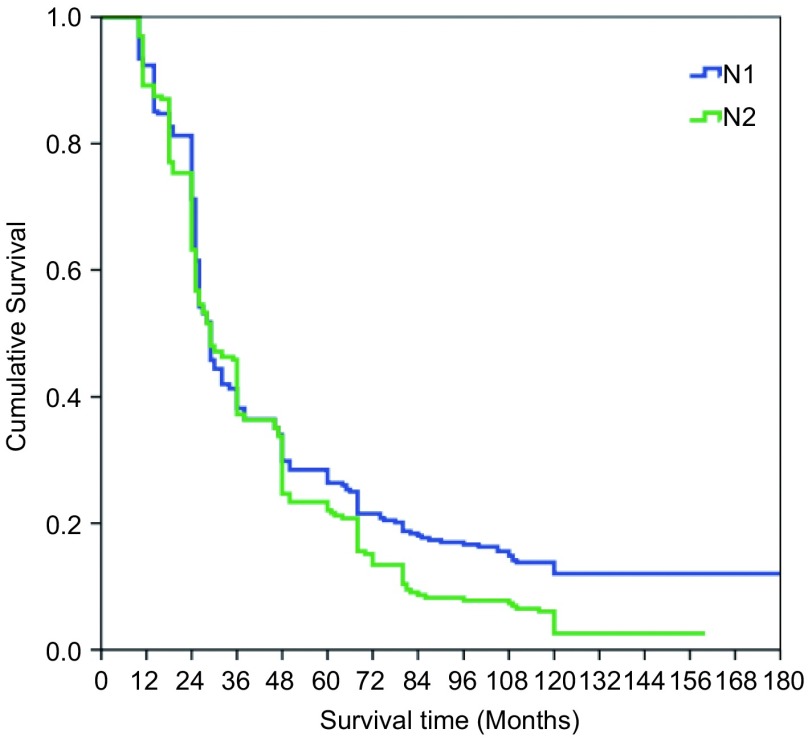
N1与N2肺癌术后生存曲线（*Kaplan-Meier*曲线）（对数秩和检验，*P* < 0.05） *Kaplan-Meier* survival curves between the patients with N1 and N2 disease (*Log-rank* test, *P* < 0.05)

**12:**
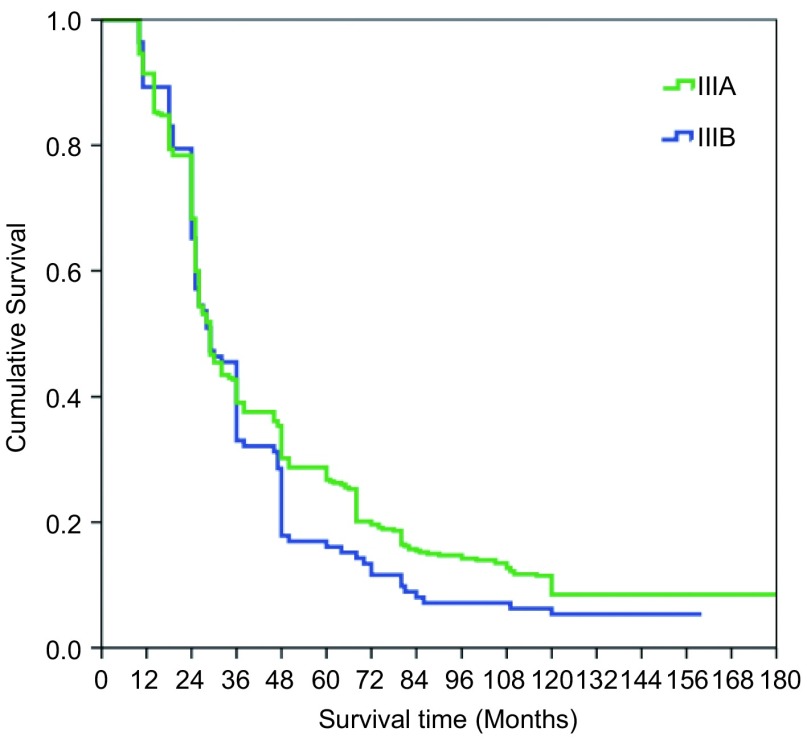
ⅢA期与ⅢB期肺癌术后生存曲线(*Kaplan-Meier*曲线)(对数秩和检验，*P* < 0.05) *Kaplan-Meier* survival curves between the patients with ⅢA and ⅢB staging (P-TNM staging) (*Log-rank* test, *P* < 0.05)

**13:**
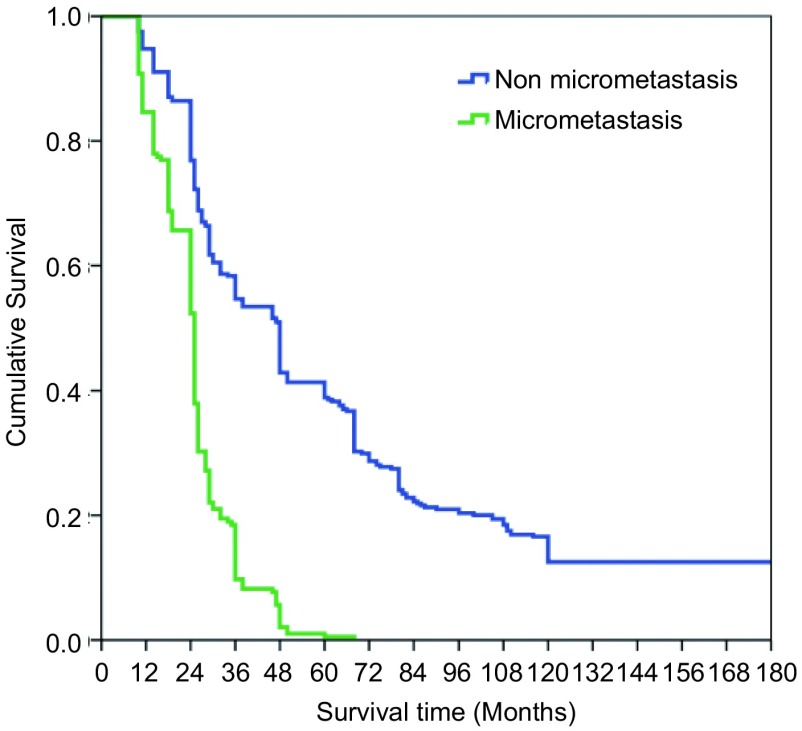
伴有与不伴有外周血微转移肺癌病人术后生存曲线（*Kaplan-Meier*曲线）（对数秩和检验，*P* < 0.05） *Kaplan-Meier* survival curves between the patients with micrometastasis and without micrometastasis in peripheral blood (*Log-rank* test, *P* < 0.05)

### 3.4 "分子分期"与肺癌病人术后生存率的关系

本研究根据肺癌病人外周血"微转移"检测结果，对肺癌进行"个体化分子P-TNM分期"，即不管病人P-TNM分期为ⅢA期，还是ⅢB期，只要外周血CK19检测阳性，均划入Ⅳ期(MP-TNM分期为Ⅳ期)。本组516例肺癌按MP-TNM分期结果如下：M-ⅢA期为109例，M-ⅢB期为266例，M-Ⅳ期为141例。M-ⅢA期肺癌病人术后1、3、5和10年生存率分别为95.5%、53.8%、40.6%和14.1%；M-ⅢB期肺癌病人术后1、3、5和10年生存率分别为89.3%、33.0%、16.1%和5.4%；M-Ⅳ期期肺癌病人术后1、3、5和10年生存率分别为63.7%、11.3%、0.7%和0.0%(图 14)。

**14:**
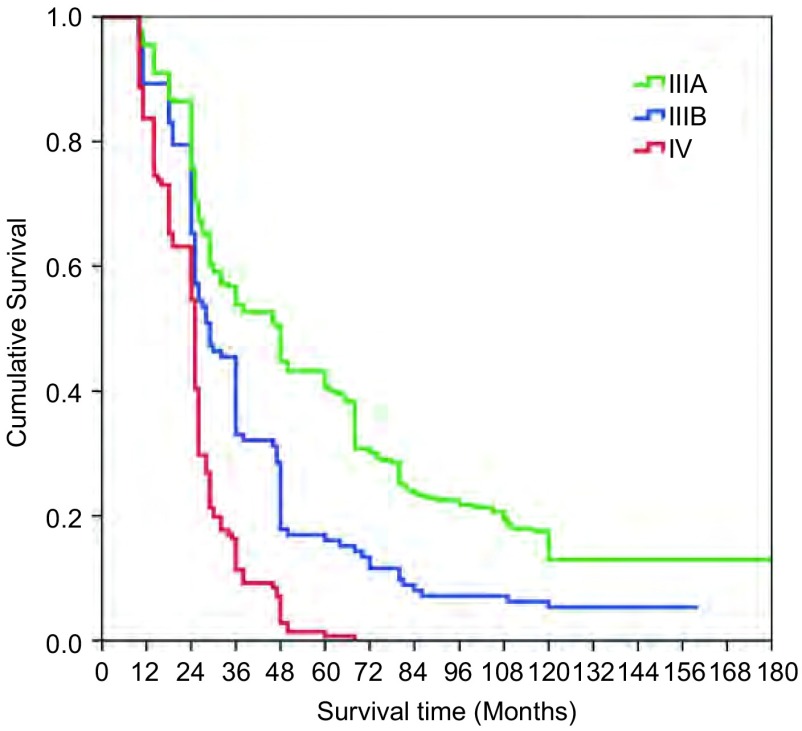
肺癌病人基于"个体化分子P-TNM"分期的生存曲线(*Kaplan-Meier*曲线)(对数秩和检验，*P* < 0.05) *Kaplan-Meier* survival curves based on personalized molecular P-TNM staging in patients with locally advanced lung cancer (*Log-rank* test, *P* < 0.05)

### 3.5 肺癌病人外周血"微转移"在预测肺癌预后中的意义

本研究应用*Cox*比例风险模型分析可能影响肺癌预后的各项因素，包括：(1)性别(Sex)；(2)年龄(Age)；(3)病理学类型(PC)；(4)吸烟(Smoking)；(5)淋巴结转移状态(N)；(6)病理分期(P-TNM)；(7)原发肿瘤大小(T)；(8)外周血"微转移" (Micrometastasis)；(9) "个体化"分子P-TNM分期(M-TNM staging) (表 4)。如表 4所示，预测肺癌预后最有意义的前四个独立因素依次为："个体化"分子P-TNM分期、外周血"微转移"、病理类型和N分期。

**4:** *Cox*比例风险模型分析结果 The result of *Cox*'s proportional hazards regression model

Items	Regression coefficients	Relative risks	*P*
Gender	-0.103	0.902	0.816
Age	-0.002	0.998	0.879
Smoke	0.01	1.01	0.391
T-staging	-0.012	0.988	0.980
P-TNM	-0.38	0.684	0.147
N-staging	-0.117	1.023	0.018
Pathological classification	0.058	1.059	0.000
Micromtastasis	0.058	1.059	0.000
M-TNM stage	0.012	1.127	0.000

## 4 讨论

### 4.1 检测肺癌微转移的分子标志物及方法

1869年，Ashworth首先报道了1例肿瘤患者外周血中存在癌细胞以来，肿瘤微转移的概念在实践中逐步得到认识并成为近年来肿瘤转移研究的热点。肺癌微转移是指播散并存活于淋巴道、血循环、骨髓及各组织器官中，采用常规检查方法，如组织病理学、影像学等难以发现的肺癌细胞团或结节，需要通过免疫学或分子生物学技术才能发现^[57-68]^。在肿瘤微转移的分子诊断研究中，标记物的含义与肿瘤标记物(tumor markers)不完全相同。前者是指正常组织细胞以及相应的肿瘤细胞所特有的，即具有组织特异性(tissue specificity)；而后者是指肿瘤组织和细胞所特有的。这类分子标记应具备2个特点：①特异性强，仅在待测的组织和来源于该组织的肿瘤细胞中表达，其它组织不表达；②敏感性高，在骨髓、淋巴结和体液中能稳定地被检测到。2001年，周清华等^[65]^报道应用RT-PCR的方法能检测到每毫升中存在10个肺癌细胞的微转移。在肺癌微转移的研究中，常用的分子标记可分为以下几类：(1)组织特异性蛋白质(tissue specific proteins, TSPs)：组织细胞内大量存在着的蛋白质是细胞生命活动的基础，蛋白质的功能各不相同。在肺癌微转移的分子诊断研究中，TSPs是应用较为普遍的标记物。组织特异性蛋白质包括以下3类：①细胞角蛋白家族(cytokeratins, CKs)：细胞角蛋白家族是检测肺癌微转移最常应用的组织特异性蛋白质。细胞角蛋白来源于上皮组织，是真核细胞的细胞骨架中间丝蛋白(intermediate filament protein)中最为复杂的一类。现在已知CK家族由分子量不等的20个成员组成，即CK1-CK20。CKs一般在上皮组织中成对表达，特异性强。CKs的阳性表达已经成为上皮细胞及其肿瘤细胞较为敏感和特异的标记。单层上皮中，所有的分泌上皮(腺上皮)均表达CK8和CK18，多数细胞还表达CK19。因此在肺癌研究中常常以CK8、CK18和CK19作为分子标记。Pantel等^[68]^以CK18为分子标记，在139例无远处转移的NSCLC患者的骨髓中检测到83例患者的骨髓微转移。②组织多肽抗原(tissue polypeptideantigen, TPA)：TPA是一种不含糖脂的蛋白质，即单链多肽。它由4个亚单位构成，其结构与CKs有较高的同源性，是CK8、CK18和CK19片段的复合物。TPA在细胞周期的S和M期合成，当细胞处于增殖分化时浓度较高。多个小组的研究^[64]^表明，TPA是肺癌患者疗效和预后的判断指标，连续检测TPA对监测肺癌的播散和肿瘤复发有较好的参考价值。③上皮特异性抗原(epithelial specificantigen, EPA)：EPA是上皮组织特异表达的蛋白质，位于细胞膜或胞浆中。目前，主要应用上皮特异性抗原癌胚抗原(CEA)和上皮膜抗原(EMA)诊断肺癌微转移。(2)组织特异性基因(tissue specific genes)：这一类基因与组织特异性蛋白质相类似，仅在上皮组织和上皮组织源的肿瘤组织中特异地表达。在最近的研究中，不少研究者以此类基因作为肺癌微转移诊断的分子标记。组织特异性基因应用于肿瘤微转移的标志物主要有以下几类：①肺泡表面蛋白mRNA：肺泡表面蛋白(surfactant proteins, SP)是肺泡表面活性物质中的主要蛋白质，由Ⅱ型肺泡细胞和Clora细胞合成分泌。在肺癌患者微转移的检测中，Betz等以SP mRNA作为标记物，检测13例M1期NSCLC患者的淋巴结中发现SP mRNA表达阳性率为84.6%(11/13)。在组织学检查阴性的淋巴结中，SP mRNA阳性检出率为55.5% (10/18)^[65, 68]^。②粘蛋白基因MUC1：粘蛋白(mucin)是一种细胞表面糖蛋白，主要存在于粘液中，由上皮组织的G细胞和某些粘膜下腺体分泌，是一类高分子量、多分散度的分子族，包括6个成员(mucin 1, 2, 3, 4, 5a, 5b)。已有报道^[59-61]^证明，MUC1在正常肺组织以及绝大多数NSCLC细胞系或实体瘤中均有较高表达，但不表达于正常的淋巴结组织中。牛中喜等^[59-61]^应用MUC1 mRNA作为分子标记，检测了119枚肺癌患者淋巴结中MUC1 mRNA表达，发现65枚存在MUC1 mRNA表达，阳性率为54.5%(65/119)；而病理方法仅检测出41枚淋巴结存在癌转移，阳性率为34.5%(41/119)。病理学检测阴性的78枚淋巴结中，经RT-PCR方法检测到24枚淋巴结存在MUC1 mRNA表达，阳性率为20.77%(24/78)。肺良性病变患者35枚淋巴结MUC1 mRNA表达均为阴性。③ CKs mRNA：CKs已经在蛋白质水平被证明是较敏感和特异的鉴别上皮组织和上皮源性肿瘤细胞的标记物之一。牛中喜等^[61]^应用CK19 mRNA检测了119枚肺癌患者淋巴结中CK19 mRNA表达，66枚淋巴结存在CK19 mRNA表达，阳性率为55.5%(66/119)，病理学方法检测出41枚淋巴结存在癌转移，阳性率为34.5%(41/119)。病理学检测阴性的78枚淋巴结中，经RT-PCR方法检测到25枚淋巴结存在CK19 mRNA表达，阳性率为32.1%(25/78)。Krismann等^[68]^研究表明以CK19 mRNA为标记物可以检测出肺癌患者周围循环中的CTCs，其阳性检出率可以达到50%(25/50)。(3)癌基因和抑癌基因：现有的研究表明，癌基因/抑癌基因的缺失、突变及过量表达是最为特异的肿瘤标志物之一。但是缺乏稳定的表达是难以以此作为肿瘤微转移分子诊断标记物的主要原因。

检测肺癌微转移的常用技术：(1)免疫组织化学技术(immunohistochemical techniques, IHC)：IHC是指以显色剂标记的特异性抗体在组织细胞原位通过抗原抗体反应和组织化学的呈色反应，对相应抗原进行定性、定位和定量的测定。在标记物抗体的选择上，多选用CK2和TPA等特异性抗体。在肺癌微转移的研究中，常用的方法主要是碱性磷酸酶-抗碱性磷酸酶法和亲合素-生物素-过氧化物酶复合物法。(2)RT-PCR：PCR是一种选择性体外扩增DNA或RNA片段的方法，即通过引物延伸核酸的某段区域而进行的重复双向的DNA合成。在肺癌微转移的研究中，应用较多的是RT-PCR技术。近年来，巢式引物(nested primer)被认为较一般引物更能避免基因组DNA扩增产物的干扰。在具体应用中，RT-PCR技术的敏感性较IHC技术高。本研究应用巢式RT-PCR检测了516例局部晚期肺癌外周血微转移，检测到114例病人外周血中存在肺癌微转移，阳性率为27.3%。(3)流式细胞术(flow cytometry, FCM)：FCM是以流式细胞仪为工具对悬液中的细胞进行检测，它采用了单克隆抗体、激光和计算机等技术，可以短时间内对大量细胞进行多参数的分析，常用于骨髓及外周血中肿瘤细胞的检测。牛中喜等^[58]^比较了FCM与RT-PCR检测同以组肺癌病人淋巴结、外周血和骨髓微转移，结果发现两组的阳性率基本一致。(4)蛋白质组学技术：蛋白质组学(proteomics)是研究细胞内全部蛋白质组成和动态变化的一门新兴学科。目前，蛋白质组学已被广泛应用于肿瘤标记物的筛选、肿瘤的发生机制、早期诊断及分类等方面的研究。Yao等^[67]^利用双向差异凝胶电泳技术联合激光捕获显微分离技术对14例肺鳞癌患者的手术标本进行蛋白质组学定量分析，结果发现10种蛋白在有淋巴结转移的患者中过量表达，4种蛋白表达减少，其中几种蛋白(Annexin A2, HSP27, CK19, 14-3-3σ)的表达与免疫印迹及免疫组化的结果一致^[67]^。(5)基因表达谱技术(gene expression profiling, GEP)：GEP是采用cDNA或寡核苷酸片段作探针，将待测样品与对照样品的mRNA以不同的荧光分子进行标记，然后同时与芯片进行杂交，通过分析两种样品与探针杂交的荧光强度的比值来检测基因表达水平的变化。它具有信息量大、操作简便及可重复性强等优点。Moriya等利用基因表达谱技术对41例肺腺癌患者进行分析，经过寡核苷酸微阵列及统计学分析，发现15种标记基因可用来检测肺腺癌的淋巴结转移，并认为分子学分期可以更准确地判断患者的预后。

### 4.2 肺癌"分子分期"在肺癌外科治疗中的临床意义

肺癌转移是肺癌的恶性标志和特征，也是导致患者死亡的直接原因。早期诊断肺癌转移，尤其是肺癌微转移，并对肺癌进行分子分期，对于肺部肿瘤外科医生选择手术适应症，指导术前新辅助治疗和术后辅助治疗的获益者，以及预测预后均有十分重要的意义。汪国文等^[66]^应用常规nested-RT-PCR获得目的基因和内参基因产物，利用微流控芯片检测，70例NSCLC患者外周血CEA mRNA表达，诊断微转移，30例肺癌病人检测到微转移。欧阳伟炜等^[69]^应用IHC检测78例Ⅰ期NSCLC根治术后区域淋巴结微转移检出率与患者长期生存的关系。报告78例P-T1-2N0肺癌784枚淋巴结CK19表达，淋巴结微转移检出率为26.9%(21/78)。不伴有微转移患者的1、3、5和8年生存率分别为90.8%、69.7%、55.76%和37.17%，中位生存期87个月；而伴有微转移患者的1、3、5和8年生存率分别为60.00%、48.48%、29.09%和0.00%，中位生存期23个月。Yamashita等^[70]^报告应用免疫组化检测117例Ⅰ期肺癌组织中VEGF-C、VEGF、E-cadherin、α-catenin、β-catenin和γ-catenin表达水平，同时检测淋巴结组织中微转移相关的标志物CK表达。肺癌组织中VEGF-C和VEGF阳性率分别为48.7%(54/117)和73.5%(86/117)。34例病理报告为N0淋巴结中检测到微转移，微转移检出率为29.1%。肺癌组织中E-cadherin、α-catenin、β-catenin和γ-catenin阳性率分别为59.8%(70/117)、35.0%(41/117)、70.9%(83/117)和52.1%(61/117)。VEGF-C在鳞癌和β-catenin阴性表达肺癌中表达尤为明显。VEGF在E-cadherin阴性表达肺癌组织中表达阳性比例高。微转移在T2肺癌和α-catenin阴性表达肺癌检出率高。单因素和多因素分析结果显示：高龄、T2肺癌和微转移是Ⅰ期肺癌术后预后不良的独立因素。Li等^[72]^应用RT-PCR检测89例P-N0 NSCLC淋巴结MUC1 mRNA表达诊断淋巴结肺癌微转移。21例病人的36枚淋巴结检测到微转移，微转移检出率为23.6%(21/89)。伴有淋巴结微转移病人的5年生存率为23.8%，而不伴有淋巴结肺癌微转移组病人的5年生存率为44.1%。多因素分析显示T分期、组织学类型和淋巴结微转移是独立的预后预测因素。Lu等^[72]^应用*meta*分析方法分析7组基因芯片检测与NSCLC生存相关差异表达基因。从7个研究中选择4, 905个基因中选择出64个基因预测Ⅰ期肺癌从术后治疗获益。*Kaplan-Meier*分析证明64个基因与生存的关系，把Ⅰ期肺癌分为高风险组和低风险组。在64个基因中，11个基因(*APC*, *CDH8*, *IL8RB*, *LY6D*, *PCDHGA12*, *DSP*, *NID*, *ENPP2*, *CCR2*, *CASP8*, *CASP10*)与肺癌术后复发转移相关。许多研究^[66-69]^已经证明：早期肺癌术后高复发转移率部分被认为与残存微转移的存在有关。

有关微转移的存在是否影响患者生存，是否需要给予额外的治疗的问题，国内外均有报道。许多研究已经证明微转移是独立预测预后不良的因素，尤其是病理诊断为N0的病人。已有的研究表明诊断微转移的检测方法对于预测微转移和生存有重要的作用。Nosotti等的研究证明应用RT-PCR检测CEA mRNA表达式预测肺癌复发最有效的方法。Hashimoto等证明伴有与不伴有微转移的P-N0肺癌之间存在显著差异。Li等的研究表明伴有淋巴结微转移的病人的生存率显著低于不伴有淋巴结微转移的病人(24% *vs* 44%)。Marchevsky等的研究证明病理诊断的P-N1肺癌的5年生存率为25%，而微转移N1肺癌的5年生存为45%。对伴有微转移的肺癌给予术后辅助治疗能降低肿瘤复发和远处转移机会，并能从术后辅助治疗获得生存获益。因此，微转移检测对于早期肺癌具有重要作用。吴等的研究也证明对于病理检测淋巴结阴性，淋巴结微转移阳性是预测病人生存的重要预测因子。Osaki等的研究表明P-N1和P-N2的病人具有更高的微转移率。Izbicki等证明不伴有淋巴结微转移的病人无复发和远程转移的生存明显高于伴有微转移的病人。已有的研究显示与7%无微转移肺癌术后发生肿瘤复发转移比较，45%伴有微转移的肺癌病人术后发生肿瘤复发转移。微转移中肿瘤复发的类型主要为血源性远处转移。已经证明单一微转移的存在预示后来发生远处转移^[73]^。Biswas等^[74]^报告了74例NSCLC(28例鳞癌，20例腺癌、9例小细胞癌、4例大细胞癌、13例未分类)骨髓微转移检测结果，74例肺癌中17例检测到骨髓微转移，检出率为23.0%。小细胞癌骨髓微转移检出率为44.4%，NSCLC为21.2%(其中大细胞癌微50%，腺癌为20%，鳞癌为17.9%)，未分类癌为15.4%。作者还发现骨髓微转移与低血小板计数间存在统计学差异(*P*=0.000, 1)。Riethdorf等^[86]^的研究表明：肺癌病人的预后是由肿瘤的远处转移决定的。在带有原发肿瘤的病人中，肿瘤的复发转移主要是由于第2个器官存在微转移，而用高分辨影像技术不能诊断。敏感和特异性的免疫化学和分子方法可以检测到骨髓和外周血中的单个播散肿瘤细胞。许多研究表明存在于骨髓和外周血中的播散性癌细胞可以抵抗化疗、处于休眠非分裂状态存活多年。Castaldo等应用RT-PCR方法检测肺癌患者的癌胚抗原mRNA，结果32例患者中29例阳性表达，其中外周血17/32(53.1%)表达。随访9个月后17例外周血表达阳性的患者有15例发生远处转移，随访24个月后15例阴性患者中只有1例发生远处转移，从而指出外周血微转移与肺癌患者的远处转移有显著相关性。Jin等^[75]^应用RT-PCR检测108例肺癌和40例良性肺病变病人，以及30例正常人外周血CK19 mRNA表达，应用ELISA检测VEGF水平，发现肺癌病人外周血VEGF为479.8±268.5 pg/mL，CK19 mRNA阳性率为66.7%，并随病理分期增高而增加。CK19 mRNA阳性病人血清VEGF为561.7±325.6 pg/mL，显著高于CK19 mRNA阴性的病人(*P* < 0.01)。肺癌病人外周血VEGF水平与CK19 mRNA阳性和患者生存间具有明显的关系(*P* < 0.01)。

为了探讨肺癌微转移"分子诊断"和"分子分期"的可行性，应用肺癌分子分期指导局部晚期肺癌外科手术适应症的选择、进行"个体化外科治疗"，以及选择术前新辅助化疗和术后辅助治疗的获益者，我们应用RT-PCR检测了516例侵犯心脏大血管的局部晚期肺癌外周血CK19 mRNA表达作为微转移分子诊断标志物来诊断肺癌微转移。本组516例肺癌病人中141例外周血检测到CK19mRNA表达，阳性率为27.3%。肺癌病人外周血微转移阳性率与肺癌组织学类型、P-TNM分期、N分期等均有密切关系(*P* < 0.05)，但与患者年龄、性别、是否吸烟、原发肿瘤大小、肿瘤部位等均无明显关系(*P* > 0.05)。本组516例肺癌病人P-TNM分期：ⅢA期112例，ⅢB期404例；全组术后中位生存时间为43.74±7.21个月，1年生存率为89.1%，3年生存率为39.3%，5年生存率为19.8%，10年生存率为10.4%。本研究根据肺癌病人外周血"微转移"检测结果，对肺癌进行"个体化分子P-TNM分期"，即不管病人P-TNM分期为ⅢA期，还是ⅢB期，只要外周血CK19检测阳性，均划入Ⅳ期(MP-TNM分期为Ⅳ期)。本组516例肺癌按MP-TNM分期结果如下：M-ⅢA期为109例，M-ⅢB期为266例，M-Ⅳ期为141例。按"个体化分子P-TNM分期"，有3例P-TNM分期为ⅢA期，138例P-TNM分期为ⅢB期的病人，"个体化分子P-TNM分期"为Ⅳ期(M-Ⅳ期)。本组M-ⅢA期肺癌病人术后1、3、5和10年生存率分别为95.5%、53.8%、40.6%和14.1%；M-ⅢB期肺癌病人术后1、3、5和10年生存率分别为89.3%、33.0%、16.1%和5.4%；M-Ⅳ期肺癌病人术后1、3、5和10年生存率分别为63.7%、11.3%、0.7%和0.0%(图 14)。*Kaplan-Meier*生存曲线结果显示：术后生存率与外周血"微转移"、肺癌组织学类型、原发肿瘤大小和淋巴结转移有密切关系(*P* < 0.05)。*Cox*比例风险模型显示"个体化分子P-TNM分期"、外周血"微转移"、病理类型和N分期是预测局部肺癌预后的独立因素。本组1例女性病人在其它医院施行开胸探查活检术，诊断为小细胞肺癌。术后E P方案化疗7周期，放疗6 5 Gy后，肿瘤进展，经临床和微转移检测证明既无临床转移，也无"微转移"，施行右全肺切除联合部分左心房、右膈肌、下腔静脉、肝右静脉切除人工血管重建。术后7年无肿瘤复发转移(图 4)。1例男性病人患右肺中心型肺癌侵犯气管隆突、右肺动脉总干和上腔静脉，伴严重的上腔静脉综合症。术前气管镜检查，病理诊断为低分化鳞癌，临床和微转移检测证明既无临床转移，也无微转移。施行支气管肺动脉袖状成形右肺中上叶切除，气管隆突切除重建，上腔静脉切除Gortex人造血管重建，病人术后存活已10年，无肿瘤复发转移。

综上所述，本研究结果表明：肺癌微转移的分子诊断敏感性、特异性强，灵敏度高，能提高淋巴结和循环系统肿瘤转移的检出率。检测肺癌微转移有助于指导外科医生选择手术适应症、选择术前新辅助化疗和术后辅助治疗的获益者。检测肺癌微转移还有助于预测患者的预后，肺癌微转移分子诊断是一个相对独立的预后指标。此外，肺癌微转移分子诊断还能为肺癌亚临床转移的逆转或分子靶向治疗提供理论基础和实验依据。因此，肺癌微转移分子诊断具有十分重要的理论和临床意义，并具有广阔的临床应用前景。

### 4.3 基于"分子分期"的肺癌"个体化外科治疗"在局部晚期肺癌多学科治疗中的地位

局部晚期肺癌，尤其是肺癌侵犯心脏大血管的局部晚期肺癌，是肺癌治疗中的一个棘手问题，无论是化疗，还是放化疗治疗疗效均不佳。而对于这类患者可否有选择地施行完全性肺切除联合受侵的心脏大血管切除，至今意见不一致。近年来，国内外学者先后报道对这类患者有选择地施行晚期性肺切除的同时，联合受侵的心脏大血管切除重建术，取得了较好的近期和远期疗效^[1-10, 41-47]^。1996年，Jeanfaive等报道对7例肺癌伴上腔静脉综合征的患者，施行肺切除合并部分上腔静脉重建术，其中1例存活5年，5例存活2年，1例存活半年以上。2001年，周清华等^[44]^报道349例侵犯心脏大血管的局部晚期肺癌外科治疗的5年生存率达到33.14%，10年生存率达到23.56%。其中肺切除合并肺动脉切除重建患者5年生存率为38.24%，上腔静脉重建术者为29.67%，左心房切除重建术者为31.23%，胸主动脉切除重建术者为33.33%。1997年，周清华等^[45]^报道肺切除合并全上腔静脉切除人造血管重建术，取得较好的临床疗效，部分患者获得长期生存。2008年，Govindan等^[3]^系统回顾分析了全世界局部晚期肺癌外科治疗的历史、现状，并展望未来发展方向，充分肯定了外科手术治疗在局部晚期肺癌治疗中的地位和取得的成果，并指出未来外科治疗仍将在局部晚期肺癌中占有更加重要的地位。此外，国内外学者在20世纪90年代先后报道了肺切除合并部分左心房切除术，其5年生存率达到25%-32%^[4, 5, 10]^。上述资料充分说明，对侵犯心脏大血管的局部晚期肺癌，只要病例选择恰当，手术能真正做到局部根治切除，同样能获得较好的近期和远期疗效。但是，临床上不少局部晚期肺癌(也包括一些IA期肺癌)术后短期内发生远处转移，并最终死于肺癌转移，表明病人在手术前就存在用传统方法不能检测到的肺癌转移，即"微转移"。因此，研究和开发能够特异性地检测到肺癌病人淋巴结、外周血和骨髓中的"微转移"，并应用微转移检测结果对肺癌病人进行"分子分期"，指导临床对局部晚期肺癌(包括早期肺癌)选择外科手术适应症，进行"个体化外科治疗"，选择术前新辅助化疗和术后辅助治疗的获益者，就成为肺癌临床研究的重要课题。我们从20世纪90年代开始应用RT-PCR技术检测肺癌病人淋巴结、外周血和骨髓中*CK19*、*MUC-1*基因mRNA表达，用于诊断肺癌"微转移"和肺癌"分子分期"，指导临床选择外科治疗适应症、术前新辅助化疗和术后辅助治疗的获益者，获得了较好的结果^[57-65]^。本文总结报告我们1999年-2003年间，应用RT-PCR方法检查516例侵犯心脏大血管的局部晚期肺癌病人外周血CK19 mRNA表达，诊断病人外周血肺癌"微转移"，对肺癌病人进行"分子分期"，指导选择外科手术适应症，并对该组病人施行"个体化外科治疗"，以及选择术前新辅助化疗和术后辅助治疗的获益者。本组516例病人术后1、3、5和10年生存率分别为89.1%、39.3%、19.8%和10.4%。其中，鳞癌组病人术后1、3、5和10年生存率分别为93.5%、48.1%、32.6%和10.4%，非鳞癌组病人术后1、3、5和10年生存率分别为86.8%、20.8%、11.2%和3.8%；不伴有肺癌"微转移"组病人术后1、3、5和10年生存率分别为94.8%、56.6%、38.9%和14.5%，伴有肺癌"微转移"组病人术后1、3、5和10年生存率分别为84.6%、9.7%、0.5%和0%。本组有13例局部晚期肺癌病人在其他医院施行开胸手术，由于肿瘤侵犯心脏大血管而未能切除肿瘤，仅行探查活检。其中1例女性病人在其它医院施行开胸探查活检术，诊断为小细胞肺癌。术后行E P方案化疗7周期，放疗6 5Gy后，肿瘤进展，经临床和微转移检测证明既无临床转移，也无"微转移"，施行右全肺切除联合部分左心房、右膈肌、下腔静脉、肝右静脉切除人工血管重建。术后7年无肿瘤复发转移(图 4)。另外，1例男性病人患右肺中心型肺癌侵犯气管隆突、右肺动脉总干和上腔静脉，伴严重的上腔静脉综合症。术前气管镜检查，病理诊断为低分化鳞癌，临床和微转移检测证明既无临床转移，也无微转移。施行支气管肺动脉袖状成形右肺中上叶切除，气管隆突切除重建，上腔静脉切除Gortex人造血管重建，病人术后存活已10年，无肿瘤复发转移。本组资料充分说明，外周血"微转移"是影响本组病人术后生存的最主要因素。因此，根据"个体化"肺癌"分子分期"，对肺癌施行"个体化外科治疗"、"个体化"术前新辅助化疗和术后辅助治疗，具有十分重要的临床意义。

侵犯心脏大血管的肺癌均为局部晚期肺癌，病情较重，全身情况较差，且很可能有潜在的远处转移，故手术适应症的选择应当十分慎重。我们经过20多年的临床研究，总结出了以下选择标准：(1)经临床检查，胸部、腹部、头部CT或MRI扫描，全身同位素骨扫描等检查，确定肺癌局限在患侧胸腔，而无双侧胸腔和远处转移者；(2)为NSCLC患者；(3)肺癌局限在一侧胸腔，无对侧肺门纵隔淋巴结转移；(4)心肺肝肾功能能够耐受拟定的手术术式；(5)不伴有癌性胸腔积液；(6)不伴有癌性心包积液；(7)肿瘤和受侵器官组织能整块完全性切除；(8)术者具有完成拟定术式的手术技能和理论知识；(9)施行手术的医院具备拟定术式的硬件和软件条件；(10)患者及家属具有承受拟定手术的心理准备，并愿意承担手术可能带来的风险；(11)最重要的是能应用分子生物学技术，检测外周血和骨髓中的微转移，进行"分子分期"，选择手术适应症，进行"个体化外科手术治疗"。本组资料充分证明了肺癌"分子分期"和"个体化外科治疗"的重要性，这也是未来肺癌外科的一个发展方向。

有关肺癌侵犯心脏大血管的患者，是先行术前新辅助化疗，还是先手术治疗，术后再行放化疗治疗，目前仍有争议。我们的经验是根据患者的不同情况采取不同的方法，即"个体化"的方法加以处理。对于原发肿瘤比较大、侵犯肺动脉的肺癌，在检测外周血微转移的基础上，原则上应先行2周期术前新辅助化疗，化疗结束后再做系统检查和微转移检测，对于既无远处转移，也没有"微转移"的病人，再施行肺切除联合受侵的组织器官完全切除。术前新辅助化疗有助于使肿瘤缩小，病期降低，消灭微转移，提高手术切除率^[43]^。对于侵及上腔静脉伴有上腔静脉综合征，或上腔静脉右心房内有癌栓形成者，进行"个体化"临床分期和"分子分期"后，对于既无远处转移，也没有"微转移"的病人，应先行手术治疗，再行术后化疗和放疗。对于侵犯左心房并伴有左心房内癌栓形成者，进行"个体化"临床分期和"分子分期"后，对于既无远处转移，也没有"微转移"的病人，应先行手术治疗，再行化疗和放疗。对于先手术治疗者，如患者一般情况好，可以考虑手术与围手术期化疗同时进行。本组大约有1/3的患者采用了手术与围术期化疗同时进行的方法，即术日麻醉开始后、手术开始前给患者施行化疗。侵及心脏大血管的局部晚期肺癌，术后均应行化疗或/和放疗。术前已行2周期化疗者，术后先行2周期化疗，然后行胸部放疗，放疗结束后再补充2周期化疗。对于术前未行化疗和围手术期化疗者，术后先化疗2周期，然后行胸部放疗，放疗结束后再行2-3周期化疗。值得注意的是：(1)凡施行过术前新辅助化疗和围术期化疗者，术后胸部放疗的剂量应当较未行化疗者低，其组织量应 < 45 Gy；(2)在化疗的同时应注意免疫支持治疗。对于施行上腔静脉切除人造血管重建术者，术后抗凝治疗尚无统一意见。我们的经验是术后前2天用潘生丁或低分子肝素治疗，拔除胸腔引流管后用华法林治疗，需终身抗凝。抗凝治疗的强度以将凝血酶原时间延长1.2倍-1.5倍为宜。

对于侵犯气管隆凸的中心型局部晚期NSCLC，施行气管隆凸切除重建符合完整切除肿瘤和尽量保留肺功能的肺癌外科治疗原则^[9-20]^。由于在麻醉管理、吻合技术以及围手术期管理等诸方面的复杂性和高难度性，气管隆凸切除重建术一直是困扰胸外科的难题，至今能开展此类手术的医院并不多。而在施行隆凸切除重建的同时，施行心脏大血管切除重建，则对胸外科医生具有更大的挑战，目前全世界仅有个别医学中心能施行这类手术^[3, 41]^。隆凸切除重建合并心脏大血管切除重建术是迄今为止胸外科领域难度最大、风险最高的手术，因此必须对每一个患者进行仔细的术前检查和充分的术前准备。术前准备包括准确的临床分期、手术耐受性的评估、手术术式和方案的设计、患者生理条件和心理条件的准备，以及与患者及家属的沟通交流等。术前检查应包括：头部增强MRI或CT扫描、胸部和腹部增强CT扫描、核素骨扫描；心肺肝肾功能测定以及对手术耐受性的评估；纤维支气管镜检查确定气管、隆凸和支气管的病变范围；将纤维支气管镜检查结果与胸部影像学检查结果、心肺肝肾功能检查结果进行综合分析判断，以便设计和确定隆凸、支气管、心脏大血管的切除范围、长度，以及气管隆凸和心脏大血管重建方式。在设计手术方案时，除需充分考虑患者因素外，还应充分考虑医院和术者自身的综合条件，是否具备施行拟定术式的能力和条件。此外，有条件的单位还应该做微转移检测，对每一个病人进行"分子分期"，并根据结果确定是否应该施行拟定的手术。

综上所述，本文首次报道通过检测516例侵犯心脏大血管的局部晚期肺癌病人外周血CK19 mRNA表达，诊断肺癌"微转移"，对肺癌进行"分子分期"，指导选择外科手术适应症，施行"个体化外科治疗"，以及选择术前新辅助化疗和术后辅助治疗的长期生存结果。本研究结果充分证明该方法具有重要的理论意义和广阔的临床应用价值，是未来肺癌外科治疗的方向，值得在临床广泛推广应用。
